# Model-free characterization of topological edge and corner states in mechanical networks

**DOI:** 10.1073/pnas.2305287121

**Published:** 2024-01-17

**Authors:** Marcelo Guzman, Xiaofei Guo, Corentin Coulais, David Carpentier, Denis Bartolo

**Affiliations:** ^a^Laboratoire de Physique, École Normale Supérieure de Lyon, Université Claude Bernard, CNRS, Lyon F-69342, France; ^b^Department of Physics and Astronomy, University of Pennsylvania, Philadelphia, PA 19104; ^c^Institute of Physics, Universiteit van Amsterdam, Amsterdam 1098 XH, The Netherlands

**Keywords:** metamaterials, topological mechanics, topological phase

## Abstract

Topological materials can host edge and corner states that are protected from disorder and material imperfections. In particular, the topological edge states of mechanical structures present unmatched opportunities for achieving robust responses in wave guiding, sensing, computation, and filtering. However, determining whether a mechanical structure is topologically nontrivial and features topologically protected modes has hitherto relied on theoretical models. This strong requirement has limited the experimental and practical significance of topological mechanics to laboratory demonstrations. Here, we introduce and validate an experimental method to detect the topologically protected zero modes of mechanical structures without resorting to any modeling step. Our practical method is based on a simple electrostatic analogy: Topological zero modes are akin to electric charges. To detect them, we identify elementary mechanical molecules and measure their chiral polarization, a recently introduced marker of topology in chiral phases. Topological zero modes are then identified as singularities of the polarization field. Our method readily applies to any mechanical structure and effectively detects the edge and corner states of regular and higher-order topological insulators. Our findings extend the reach of chiral topological phases beyond designer materials and allow their direct experimental investigation.

## Significance

Topological mechanics has emerged as an effective design strategy of structures that can steer stresses and deformations for vibration control, wave guiding, and robotics. However, the concept of topological phases is rather abstract, and the characterization of the spectral topology of mechanical structures has intrinsically relied on the a priori knowledge of idealized theoretical models. Here, we introduce a fully experimental strategy to detect and locate the topological soft and weak spots of mechanical structures without resorting to any theoretical or numerical model. We expect this insight into topological mechanics to make it accessible to a broader range of physicists and engineers and to open guidelines to construct genuine functional materials beyond laboratory demonstrators.

“To measure is to know.” This century-old tenet traced back to Lord Kelvin (1) has been foundational in the discovery of material properties. The ancient Greeks discovered magnetism long before the concepts of magnetic moment and spin were introduced (2), Hooke discovered the laws of elasticity before the introduction of tensorial mechanics (3), and the quantized conductance of the quantum Hall effect was discovered prior to the concepts of topological phases (4, 5). In this article, we follow Kelvin’s wisdom, and we experimentally reveal the topological phases of mechanical metamaterials and locate their soft and weak spots without resorting to any a priori modeling.

This insight into structural mechanics (6, 7) is atypical from the perspective of metamaterial science. Metamaterials are designed (6, 7). It is only after a necessary modeling step that they can be engineered and eventually achieve properties out of reach of conventional matter. In particular, the field of topological mechanics has bloomed since the identification of a formal correspondence between the linear dynamics of isostatic lattices of beads and springs and tight binding models of topological insulators in quantum matter (8–10). This correspondence then led to a new stream of design strategies for mechanical structures featuring polar mechanical response (11–13) or directional phononic wave channels (14, 15). Today, virtually all types of electronic topological phases have found a mechanical counterpart, from the topological insulators and superconductors of the tenfold classification (16), to non-Hermitian (17, 18), quantum spin-Hall (19), and even higher-order (20–23) topological phases.

**Fig. 1.**
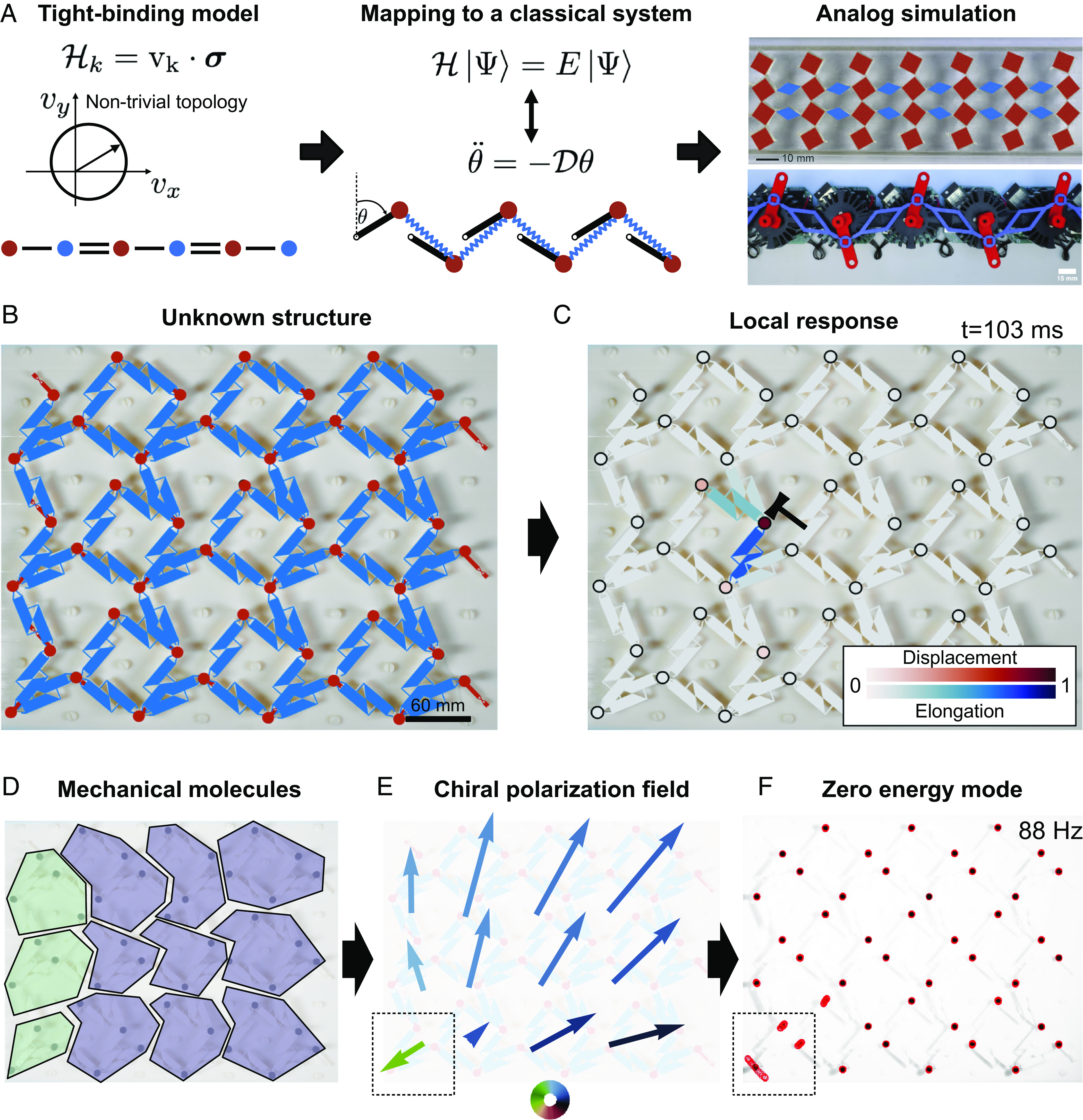
Two complementary perspectives on topological Mechanics. (*A*) Topological metamaterial design. The starting point is the tight binding Hamiltonian of a quantum electronic system. We give the example of a 1D chiral Hamiltonian $H_{k}=v_{k}^{i}\cdot \sigma^{i}$, where the $\sigma^{i}$ are two Pauli matrices and v a real 2D vector indexed by the wave vector *k*. The Hamiltonian is characterized by a topological index. Here, given the unit cell sketched in the figure, H is associated with a nonvanishing winding number, the topological index of chiral Hamiltonians, given by the winding of $v_{k}$ around the origin as k varies. The Hamiltonian dynamics is then mapped on the classical mechanics of mechanically coupled structures such as a network of beads and springs. The bead and spring network can then be approximately realized with a variety of materials. The *Upper Right* panel shows a classical realization of the celebrated SSH Hamiltonian (13), while the *Bottom Right* picture shows a realization of its non-Hermitian counterparts (17). (*B*) Model-free detection of topological zero modes in unknown mechanical structures. The starting point is now an a priori unknown mechanical structure made of passive units such as connected beads, springs, and beams. (*C*) We identify both the displacement and elongation degrees of freedom and measure their dynamic response to a point force. The blue and red colormaps indicate the instantaneous magnitude of the displacements and elongations in response to a local point force; see *Materials and Methods*, *SI Appendix* and Fig. 2 for more details. (*D*) and (*E*) The weighted average of the displacements and elongations allows us to define mechanical molecules and their associated local chiral polarization Π(r), which generalizes the concept of topological polarization introduced in ref. 8.The big bulk molecules are highlighted in violet while the smallest, belonging to the left edge, in green. (*F*) Assemblies of mechanical molecules that share a chiral polarization pointing along the same direction feature the same spectral topology. Conversely, singularities (i.e., discontinuities of the order of the lattice spacing) in the polarization field reveal the existence of localized zero energy modes: floppy modes and states of self-stress. Here, the discontinuity is localized at the *Bottom Left* corner (dashed square) and the presence of a corner floppy mode is verified by shaking the whole sample at the lowest eigenfrequency: 88 Hz. The red circles track the positions of the beads over time.

These diverse realizations all stem from the same design protocol, which relies on rather abstract concepts. We summarize it in Fig. 1*A*. The starting point is a tight binding Hamiltonian H known to feature a so-called nontrivial topological index (24). H typically describes the quantum dynamics of electrons in a solid. The next step consists in using a formal mapping from the quantum dynamics ruled by H to a classical Newtonian system (8, 16). Finally, a mechanical structure is designed and engineered to perform an analog simulation of the classical dynamics. The key property of the resulting structures is the existence of edge states protected by the nontrivial topology of the bulk-vibration modes, a consequence of the bulk-boundary correspondence principle.

In this article, we take an alternative perspective on topological mechanics. We place measurements as a primary tool to inquire about the properties of mechanical structures. We aim at answering a basic question: Given a rigid structure (isostatic or hyperstatic) assembled from beads, springs, or beams, can one predict the existence and location of its topologically protected floppy, or self-stress, modes without resorting to any theoretical model? The motivation is clear as these two modes can either offer functional capabilities or limit the range of applications of a mechanical structure. In mechanical insulators, floppy modes are localized soft spots which respond nonlinearly to vanishingly small forces (12, 25–28), while self-stresses are weak spots precursor of failure upon external loads and cannot be detected from deformations fields (27, 29–31).

To predict and locate the soft and weak spots in natural or man-made structures, we introduce a generic method inspired by classical electrostatics (7). We illustrate it in Fig. 1*B*–*F*. Starting from an unknown structure, we poke it locally and measure the resulting displacements and deformations, Fig. 1*C*. From the spatial correlations between the deformation and displacement fields, we then identify mechanical “molecules” (Fig. 1*D*). They are defined as the most strongly coupled displacement and deformation degrees of freedom. From the measured responses, we can then define a polarization Π for each molecule (Fig. 1*E*). This so-called chiral polarization encodes both the local rigidity and spectral topology of the metamaterial and was theoretically defined in ref. 32. Π(r) is a local material property that generalizes earlier macroscopic markers of topological phase models: the topological polarization introduced by Kane and Lubensky (8), the topological contribution to the polarization of electronic insulators (33), and the mean chiral displacement of photonic metamaterials (34, 35). We explain how to measure Π and show that floppy and self-stress modes correspond to net mechanical charges signaled by topological defects in the chiral polarization field (Fig. 1*F*). Our minimal method predicts the location of the zero-energy modes without resorting to any theoretical model or abstract topological concepts. Combining experiments, simulations, and theory, we establish the robustness of our predictions to uncontrolled dissipation processes, nonlinearities, and material imperfections.

## Method: Basic Concepts and Tutorial Example

### Poking the Mechanical SSH Chain.

**Fig. 2.**
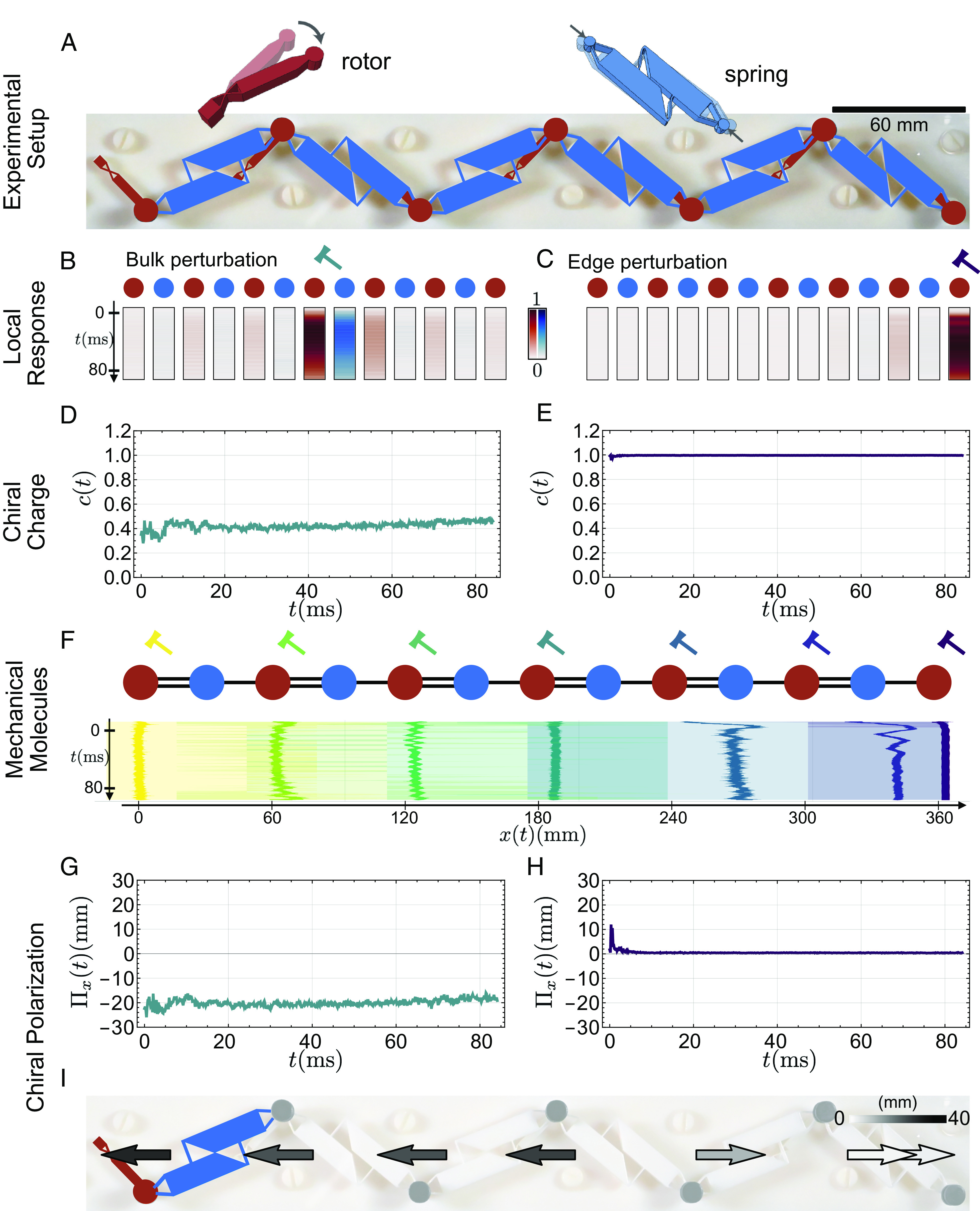
Mechanical molecules and chiral polarization in a 1D mechanical insulator. (*A*) A 3D-printed mechanical chain assembled from seven rotors (red) connected by springs (blue). The system hosts a floppy mode (FM) in the rightmost node (*Materials and Methods*). (*B*) Chiral representation of beads (red circles) and springs (blue circles). We locally excite the fourth node of the sample by poking the bead with a hammer. The response of the elongation and displacement degrees of freedom propagate in time and space (experimental measurements). (*C*) In contrast, poking the last node results in a localized response where almost no elongation is observed. (*D*) To account for the nature of the response, whether it is displacement or elongation dominated, we trace its chiral charge. The bulk response is characterized by an oscillating charge around c=0.4. (*E*) In contrast, the rightmost perturbation is characterized by c=1 typical of a floppy mode. (*F*) The response, as seen in panels *B* and *C* remains centered around the excited node. Its spatial extent is captured, for example, by the CI of the probability distribution defined by Ψ, here set to 95%. The mechanical molecules are confined regions in space of size comparable with the length-scale of the repeating motif (*Bottom*). (*G, H*, and *I*) We assign the chiral polarization Π(t) to each mechanical molecule. It is a vector which characterizes the (chiral) topological phase of the system. The source-like discontinuity of the field signals the floppy mode. In practice, a discontinuity of the chiral polarization corresponds to a change of one of its coordinates of the order of the lattice spacing a=60 mm.

For the sake of clarity, we first explain and benchmark our method using a tutorial example. We consider the structure shown in Fig. 2*A*. It is a compliant realization of the Kane–Lubensky chain first introduced in ref. 8. This 3D-printed structure is made of repeated units hosting one angular degree of freedom (the bead position shown in red), and one spring (shown in blue). When the rest angle $\theta_{0}$ is nonzero, the isostatic metamaterial is a mechanical insulator (8, 36). Fig. 2 *B* and *C* show that when we locally poke one bead with a hammer, the response remains localized in space; see also methods and *SI Appendix* where we provide a detailed description of the experiments. This seemingly mundane operation reveals two essential features: First, when hitting a bead, the neighboring springs evolve their elongation, but do not respond symmetrically: The bulk response of the metamaterial is intrinsically polar, Fig. 2*B*. Second, when poking the rightmost bead, the elongation of the neighboring springs remains unchanged; they therefore come at no elastic energy cost and define a floppy mode (Fig. 2*C*). We now make our observations more quantitative. Our goal is to i) experimentally identify the topological character of the metamaterial spectrum based on our measurements only and ii) predict the nature and location of the topologically protected zero-energy modes with a minimal sampling protocol. This protocol holds significant importance for self-stress modes, which present a greater challenge for detection compared to floppy modes. As a matter of fact, a self-stress is a mechanical state where finite stresses result in no forces and hence in no bead dynamics.

Our method fundamentally relies on the concepts of chiral polarization, and more broadly on chiral symmetry, a generic feature of mechanical networks. These two concepts were both thoroughly discussed in ref. 32 from a sole theoretical perspective. In the remainder of the manuscript, we build on these concepts to introduce a model-free characterization of topological edge and corner modes.

### Chiral Symmetry.

All mechanical structures can be described as assemblies of beads connected by springs. This elementary observation translates in a fundamental symmetry: chiral symmetry.^*^ We now show the practical relevance of this seemingly formal observation. In short, we note u(t) the vector formed by the displacements of all beads, and e(t) the displacements of all springs. The two quantities are linearly related via the connectivity of the mechanical network: $e=Q^{T}u$, where $Q^{T}$ is the so-called compatibility matrix (26). When the spring response is linear, the elastic energy modes are identical to the spectrum of the matrix:

[1] $$\begin{array}{c} H=\left(\begin{array}{cc} 0 & Q\\ Q^{\;\text{T}} & 0 \end{array}\right)\;. \end{array}$$

From a condensed matter perspective, H would be the Hamiltonian a free quantum particle evolving on a bipartite lattice defined by the bead and spring positions, respectively, red and blue sites in Fig. 2*B*. Crucially, the lattice connects only blue sites to red sites and vice versa. There is no connection between sites of identical nature.

This simple geometrical property formally translates to chiral symmetry: H anticommutes with the diagonal chiral operator: HC=−CH, where $C=\left(\begin{array}{cc} 1 & 0\\ 0 & -1 \end{array}\right)$. To see how this symmetry rules the dynamics of the network, we recall that the Schrödinger equation defined by H describes the dynamics of the wave function $|\Psi (t)\rangle =\left(u(t),e(t)\right)$ which encodes the full information about both the displacements and elongations in the network (26) (Units are chosen so that the bead mass and spring stiffness are both equal to one). We explicitly recall in *SI Appendix* how to map the Newtonian dynamics of the assembly of beads and springs on the above Shrödinger picture.

### Chiral Charge.

The structure of C suggests an intuitive electrostatic analogy. We can assign a +1 charge to the displacement degrees of freedom and a −1 charge to the spring deformations. This analogy is useful to quantify the nature of the response to a mechanical perturbation. When poked, a mechanical structure undergoes vibrations, where the beads move and the springs deform. The presence of asymmetric lever arms—always at stake in topological Maxwell lattices—implies an imbalance between deformations and displacements. To quantify this imbalance on the response to a perturbation applied to the ith bead, we define the chiral charge $c_{i}\left(t\right)$ as:

[2] $$\begin{array}{c} c_{i}\left(t\right)=\frac{u^{\text{T}}u-e^{\text{T}}e}{u^{\text{T}}u+e^{\text{T}}e}=\frac{\left\langle \Psi |C|\Psi \right\rangle }{\left\langle \Psi |\Psi \right\rangle }. \end{array}$$

Positive (resp. negative) chiral charges indicate the preponderance of displacements (resp. stresses), the extreme cases being floppy modes (c=1) and states of self-stress (c=−1) as exemplified by our experiments in Fig. 2*D*. In electrostatics, charges are bound to molecules. To pursue the analogy further, we therefore need to define “mechanical molecules.”

### Mechanical Molecules.

We now define the “mechanical molecules” hosting the chiral charges: the ensemble of beads and springs that are the more strongly coupled and separated by less than one lattice spacing. To do so, we hammer all the rotors of the chain one at a time. We then track the bead displacements u(t) and spring elongations e(t). Our experiments show that the resulting wave function Ψ(t) is i) localized in space and ii) asymmetrically distributed around the bead that has been perturbed. In the experiment shown in Fig. 2*B*, the shape of Ψ(t) gives a visual indication of which beads and springs are the most strongly coupled, thereby defining what we dub a “mechanical molecule.” This definition can be made more systematic. In all that follows, we experimentally define a molecule by the ensemble of beads and springs within the region of space that corresponds to the 95% CI associated with the distribution Ψ(t) (Fig. 2*F* and *Materials and Methods*).

Intuitively, the pairs of beads and springs that form the molecules are those that are coupled by the largest lever arm: In our experiments, when a bead moves, it primarily deforms the spring sitting on its right-hand side. In the language of topological insulators, the mechanical, or chiral, molecules define the unit cells compatible with the atomic-limit of the linearized Hamiltonian H (32, 37). In mechanics, the atomic limit is reached when the lever arm between two molecules is vanishingly small, i.e., when the rotor and the spring are colinear; see *SI Appendix* for more examples.

We finally introduce the center of the mechanical molecules as the time average of the instantaneous weighted position $r_{i}\left(t\right)$ defined by:

[3] $$\begin{array}{c} r_{i}\left(t\right)=\frac{u^{\text{T}}R_{u}u+e^{\text{T}}R_{e}e}{u^{\text{T}}u+e^{\text{T}}e}=\frac{\left\langle \Psi |R|\Psi \right\rangle }{\left\langle \Psi |\Psi \right\rangle }. \end{array}$$

$R_{u}$ (resp. $R_{e}$) is a diagonal matrix whose entries are the equilibrium coordinates of the beads (resp. springs), and R is the combined position operator.

### Chiral Polarization.

In the Kane–Lubensky chain, as in all Maxwell lattices, the molecules include as many beads as springs. The molecule is therefore “geometrically neutral.” However, the structure shown in Fig. 2*F* is not tesselated by an integer number of neutral molecules. This observation suggests that the rightmost edge of the structure might host a net +1 chiral charge, i.e., a floppy mode. It corresponds to a bead whose displacement is not coupled to spring elongations.

Our electrostatic analogy then begs for defining another material property: a mechanical polarization. Just as one would define an electrostatic dipole, we can define locally a polarization vector Π(t) as the response function to a perturbation applied to the ith bead:

[4] $$\begin{array}{cc} \Pi_{i}\left(t\right) & =2\left(\frac{u^{\text{T}}R_{u}u-e^{\text{T}}R_{e}e}{u^{\text{T}}u+e^{\text{T}}e}-c_{i}\left(t\right)r_{i}\left(t\right)\right)\\ & =2\left(\frac{\left\langle \Psi (t)|CR|\Psi (t)\right\rangle }{\left\langle \Psi (t)|\Psi (t)\right\rangle }-c_{i}\left(t\right)r_{i}\left(t\right)\right). \end{array}$$

This quantity is nothing else but the chiral polarization introduced in ref. 32. In Fig. 2*G*, we plot the *x*-component of the time-averaged chiral polarization measured in our experiments. Away from the floppy mode ${\left\langle \Pi \left(t\right)\right\rangle }_{t}$ is uniform and points toward the left-hand side. In simple terms, the polarization $\Pi_{i}$ always points from the poked bead to the spring whose deformation is the most prominent due to the lever effect. This is the reason why chains with right-leaning and left-leaning rotors have opposite polarizations, as we will see in the next section.

However, close to the right edge of the sample, the chiral polarization field is singular and changes sign. This singularity coincides with the location of the floppy mode, Fig. 2*C*. We show below that this observation has a fundamental origin. It is deeply rooted in the bulk boundary correspondence of topological insulators. To explain it, we first need to relate the measure of $\Pi_{i}$ to the topological index of chiral topological phases.

In ref. 32, we showed that, in the bulk of a periodic Maxwell lattice whose dynamics is described by a Bloch Hamiltonian H(k),

[5] $$\begin{array}{c} \Pi_{i}=p-aw, \end{array}$$

where *w* is the winding number of H(k), and *p* is the geometrical polarization of the unit cell of size *a* used to define the Bloch Hamiltonian. Strictly speaking, this formula applies when the initial perturbation excites a so-called Wannier state of H (37), a condition which is in principle never met in our experiments. However, we numerically show in *SI Appendix* that Eqs. **4** and **5** lead to the same measure of $\Pi_{i}$, which hardly depends on the specifics of the poking protocol (provided that it is localized in space). At long times, $\Pi_{i}\left(t\right)$ weakly fluctuates around a well-defined average value for ${\left\langle \Pi \left(t\right)\right\rangle }_{t}$. This numerical result implies that the measurement of a net chiral polarization field in an experiment unambiguously signals a nontrivial bulk topology. We can draw three conclusions from this central result:

(i) In crystalline lattices, away from the edge, translation invariance implies that the chiral polarization field is homogeneous. From a single poking experiment, we can then measure the bulk chiral polarization and therefore deduce the topological nature of the metamaterial without resorting to any theoretical model.
(ii) In our experiments, the 3D-printed structures dissipate energy. In addition, by hammering them, we excite a variety of structural modes which are not captured by the naive bead and spring model we use to predict the value of Π. Nonetheless, all our results are in excellent agreement with the prediction of the chiral polarization based on the Kane–Lubensky model with no free-fitting parameter. We measure $\Pi_{x}=21.5\pm 1.2$ mm and our linear theory predicts $\Pi_{x}=30\pm 6$ mm (see Fig. 7 in *Materials and Methods*). The other two options for the model are either $\Pi_{x}=0$ mm corresponding to a conductor or $\Pi_{x}=-30$ mm corresponding to an insulator with opposite polarization; *SI Appendix*. This agreement echoes the strong robustness of our method to geometrical, material, and protocol imperfections.
(iii) Eq. **4** does not rely on any underlying periodic structure or any assumption about the isostaticity of the mechanical structure. In the remainder of the article, we demonstrate that this method further applies to heterogeneous and hyperstatic structures.

### Bulk Boundary Correspondence and Optimal Detection of Zero Energy Modes.

In principle, the detection of floppy modes and states of self-stress in an unknown sample requires measuring the displacement and stress response everywhere in space and at all frequencies. Here, we show how to circumvent this costly procedure.

To do so, we build on the relation between $\Pi_{i}\left(t\right)$ and the winding number of a chiral Hamiltonian to take advantage of the bulk-boundary-correspondence principle. This principle relates the bulk topology of a material to the existence of edge states (26, 32, 38). Topologically protected zero modes exist when (32): i) the chiral polarization in the bulk does not vanish, and ii) the metamaterial is not composed of an integer number of mechanical molecules. This last condition equivalently means that the lattice cannot be tesselated with unit cells compatible with the atomic limit of H (32). As conjectured above, this situation is analogous to a molecular chain hosting dielectric dipoles. When a chain includes an integer number of molecules the overall charge vanishes. Conversely when a fraction of a molecule is added at its end, the chain hosts an additional uncompensated charge. The sign of the charge is then determined by the orientation of the electric dipole.

We can use this analogy to locate mechanical zero modes in the structure shown in Fig. 2*A*. First, we note that it cannot be fully tesselated with mechanical molecules; in Fig. 2*E*, we see that an extra bead must be added on the right-hand side to complete the chain. Second, ${\left\langle \Pi \left(t\right)\right\rangle }_{t}$ is finite and points toward the left-hand side in the bulk. Altogether, these observations tell us that a floppy mode (positive chiral charge) must exist at the rightmost end of the chain, as confirmed by our observations and measurements, Fig. 2 *C* and *E*. We stress that our method requires poking a single bead in the bulk to predict and locate a floppy boundary mode.

**Fig. 3.**
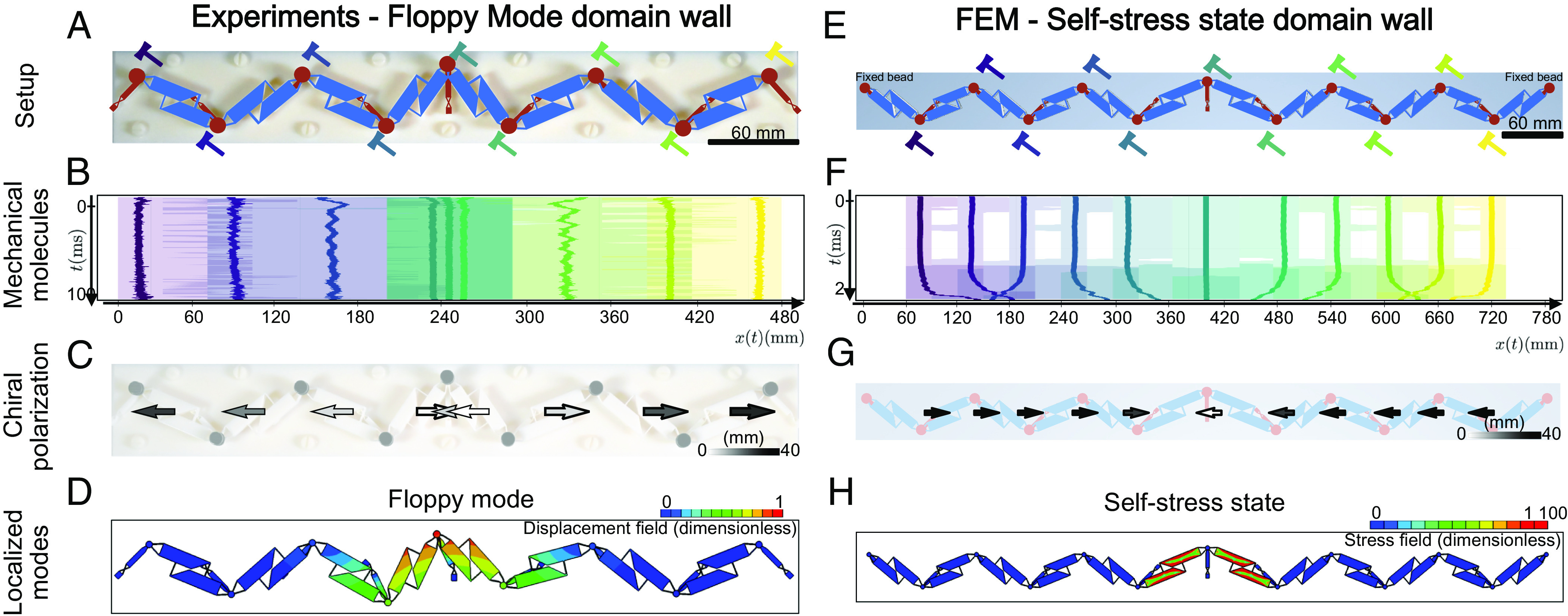
Detecting topologically protected zero modes from poking experiments. (*A*) 3D-printed mechanical chain with an angular discontinuity at the central rotor. We locally perturb each node by poking them with a hammer. Each perturbation corresponds to a different color. (*B*) To characterize the response to the nine poking experiments, we track the average positions $r_{i}\left(t\right)$ (solid line), and the extent of the associated chiral molecules defined by the 95% CI associated with Ψ (shaded regions). Away from the structural discontinuity, the molecules include one bead and one spring. At the boundary between the left and right domains, the chiral molecules overlap. (*C*) Average chiral polarization field, located at the average centers of perturbations. The arrows are shown with a fixed length of 30 mm and the magnitude is depicted with a grayscale. From *Left* to *Right*, the polarization switches sign from negative to positive. The midpoint is characterized by a discontinuity in the direction of the field. The source of the discontinuity indicates the presence of the floppy mode. (*D*) The prediction is confirmed by FEM simulations. Here we show the lowest energy mode of the system. It corresponds to a localized displacement mode, i.e., a floppy mode localized at the boundary between the two topologically distinct Kane–Lubensky chains. (*E*) Using FEM simulations we analyze the complementary version of the system shown in *A*, where left and right sides are swapped. Moreover, we fix both ending beads in order to avoid the edge floppy modes. (*F*) From each perturbation, implemented by imposing local displacements on the nodes at t=0, we track the center and the extent of the mechanical molecules (CI of 95%). Due to the absence of damping in the simulations, the perturbations do not fade away. They instead bounce back and forth in the finite system. We therefore consider the evolution of the molecules in a small time window of 2 ms. (*G*) The resulting chiral polarization field displays a sink-like discontinuity at the boundary between the two distinct domains. This observation is consistent with the existence of a topological state of self-stress (*H*) FEM simulations.

To further confirm the predictive power of our method, we performed additional experiments and finite element method (FEM) simulations. We first consider the heterogeneous metamaterials shown in Fig. 3*A*. It is composed of two Kane–Lubensky chains leaning in opposite directions. We use the same procedure as above to experimentally identify the mechanical molecules Fig. 3*B*, and their chiral polarization, Fig. 3*C*. We find again that we cannot tesselate the full metamaterial with a single type of mechanical molecule and that the chiral polarizations of the two domains leaning in opposite directions have opposite signs, Fig. 3*C*. The ${\left\langle \Pi \left(r,t\right)\right\rangle }_{t}$ field has a positive singular divergence at the boundary between the two domains. More specifically, the jump in the *x* component of ${\left\langle \Pi \left(t\right)\right\rangle }_{t}$ is of the order of one half of a unit cell *a*: $|\Pi_{x}^{\text{left}}-\Pi_{x}^{\text{right}}|\approx 0.77a$. These observations imply that a topologically protected floppy mode must exist at the junction between the two domains. The first eigenmode of the FEM simulation unambiguously confirms this prediction Fig. 3*D*.

Similarly, we perform a FEM analysis of the second heterogeneous structure shown in Fig. 3*E*. In this case, the Π field has a negative singular divergence. ($|\Pi_{x}^{\text{left}}-\Pi_{x}^{\text{right}}|\approx 0.79a$), Fig. 3*G*. We therefore expect the localization of a state of self-stress at the boundary between the two domains, as demonstrated in ref. 8. It is worth noting that self-stress states cannot be directly detected from linear displacement modes. However, here, we can numerically track them thanks to the sub-unit resolution of the FEM simulations for the lowest energy mode, Fig. 3*H*, confirming our prediction.

To conclude, we stress the efficiency of our method. When a metamaterial is made of a polycrystalline structure, only one poking experiment per domain is enough to predict the existence and the nature of localized zero modes. One poking experiment in the bulk of each domain gives information about the orientation of the chiral polarization of the corresponding crystal. Any discontinuity in the polarization of the order of, or larger than, one lattice spacing between two domains implies the existence of topologically protected zero modes at the boundary. This procedure offers a complementary approach to traditional modeling processes, relying on the computation of topological invariants or the decay rates of the eigenmodes (39), especially when dealing with complex or poorly understood systems. Our method is also more efficient than any experimental protocol based on a full spectral characterization, which requires an extensive number of independent measurements; *Materials and Methods* and *SI Appendix*.

## Zero Modes and Topological Phases of Higher Dimensional Metamaterials

Our model-free characterization of topologically polarized metamaterials has so far been limited to a canonical case where each mechanical molecule is made from a single pair of atoms. However, most mechanical structures have multiple degrees of freedom per unit cell. They are made of “polyatomic mechanical molecules,” or in the language of band theory, each unit cell includes several Wannier centers. The strategy to detect topologically protected zero modes remains the same: i) We poke the system to identify the mechanical molecules; ii) we define their chiral polarization; and iii) if the material cannot be tesselated with an integer number of mechanical molecules, we look for discontinuities in the polarization field and hence resolve the location of states of self-stress and floppy modes.

**Fig. 4.**
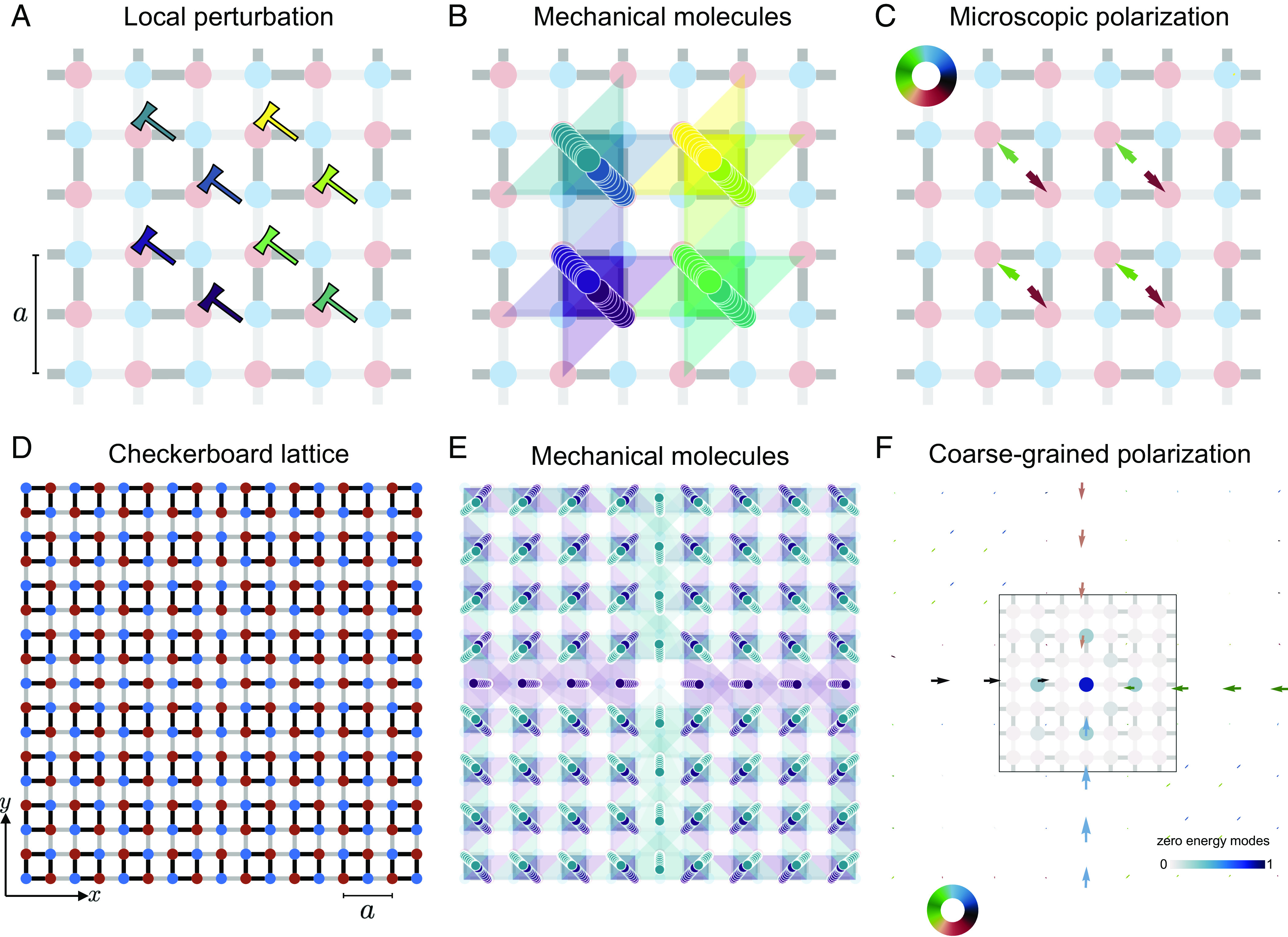
Polyatomic mechanical molecules. (*A*) Model-free characterization of the checkerboard lattice through local perturbations (hammers). The chiral network connects degrees of freedom (red disks) to constraints (blue disks). The unit-cell length is *a*. (*B*) The centers of the perturbations r(t) jiggle around confined regions with periodicity a. Unlike the 1D example, here two centers revolve around the same area, they both define the mechanical molecule. This is further confirmed by the spatial spreading of the perturbations (colored polygons). it is defined through the Mahalanobis distance computed from Ψ(t) (*Materials and Methods*). Here, we superimpose the polygons for each time step. The opaque regions where the perturbations overlap include the two beads and two springs that form the mechanical molecule. (*C*) Each perturbation leads to a time-average polarization, illustrated by the arrows. The polarization field is highly discontinuous at the microscopic scale. However, it vanishes when coarse-graining over the mechanical molecules. (*D*) Finite size checkerboard lattice of 17 × 17 nodes. (*E*) We simulate the evolution of local perturbations over all the degrees of freedom, as in *A*. To reveal the spatial arrangement of the mechanical molecules, we superimpose the colored polygons as in *B*. The outcome clearly distinguishes two kinds of molecules: complex molecules of two degrees of freedom and two constraints, and simple molecules of one degree of freedom and one constraint. (*F*) Coarse-graining the field unveils four sectors of null polarization connected by finite domains of finite polarization. Furthermore, the field has a sink-like discontinuity at the center; the system hosts a localized self-stress state.

To clearly demonstrate this generalization, we first build on the well-known theoretical model sketched in Fig. 4*A*, which we characterize numerically. This model is a paradigmatic example of a higher-order topological insulator; it consists of a chiral tight-binding Hamiltonian defined on the checkerboard lattice (40) and maps onto the dynamics of the mechanical structure discussed in ref. 41.

### Polyatomic Mechanical Molecules.

The checkerboard lattice includes two degrees of freedom and therefore two Wannier centers, per unit cell. The identification of the beads and springs that define the mechanical molecules hence requires an extra step. Without the a priori knowledge of the bond strength, we need to study the response to more than two local perturbations to define our mechanical molecules, Fig. 4*A*. Fig. 4*B* shows the time evolution of the trajectories of the centers $r_{n}\left(t\right)$; Eq. **3** and the spreading of Ψ(t) in response to localized perturbations. We can then readily generalize the method introduced in the previous section, and define the mechanical molecule as shown in Fig. 4*B*.

### Chiral Polarization Texture.

We can now define the chiral polarization associated with the two beads poked within the mechanical molecules. We use the same definition as in Eq. **4**. Fig. 4*C* reveals that each mechanical molecule features an inner polarization texture where the two beads are associated with opposite chiral polarizations. In all that follows, we disregard this microscopic structure reminiscent of the quadrupolar order discussed at large in ref. 40. We instead define the total chiral polarization by computing

[6] $$\begin{array}{c} \Pi =\sum_{n=1}^{N}{\left\langle \Pi_{n}\left(t\right)\right\rangle }_{t}, \end{array}$$

where *N* is the number of degrees of freedom per mechanical molecule.

### Detection of Topologically Protected Corner States.

In Fig. 4*D*, we show a structure assembled from four different checkerboard lattices. We find that the whole structure cannot be tesselated by a single mechanical molecule. It is instead cut into four quadrants by two perpendicular lines of “half-molecules” (Fig. 4*E*).

Fig. 4*F* shows the full chiral polarization field. This pattern allows us to make two central predictions. First, the structure is topologically heterogeneous. The four quadrants are topologically trivial as far as the chiral polarization is concerned the structure is isostatic and Π is vanishingly small (32). There is no discontinuity of the chiral polarization in the directions normal to the edges of the sample or to the domain walls. This observation implies that the metamaterial does not feature any topological edge state. However, the cross-shaped domain wall forms 1D topological insulators with incompatible chiral polarizations. We therefore predict the existence of a zero energy mode at their intersection. As the polarization field points toward the center of cross, it must host a negative chiral charge, i.e., a state of self-stress. This prediction is in perfect agreement with the distribution of the zero energy eigenmode found by diagonalizing H and plotted in Fig. 4*F*. It is worth noting that we have detected the topologically protected zero mode at the intersection between four higher-order topological phases without resorting to any higher-order topological invariant and without exploiting the fourfold symmetry of the lattice (40, 41). Our prediction solely stems from chiral symmetry, or more accurately from the incommensurability of the system with the mechanical molecules, and from the existence of a nonvanishing chiral polarization along the edges.

This last step concludes the presentation of our method. We are now ready to apply it to actual metamaterials which are not mere analog simulations of well-established models.

## Results

We apply our method to predict the existence and locate the topologically protected zero modes of the 3D-printed structure shown in our first figure, Fig. 1*B*. This metamaterial is a piece of a crystal assembled from repeated units including 6 springs (blue) and 4 beads (red). Three beads are located at the end of rotors and are therefore associated with a single degree of freedom. We know from the Maxwell–Calladine count that the structure is hyperstatic as the number of degrees of freedom (5) is smaller than the number of springs (6). Each unit cell therefore features one state of self-stress. Our goal is now to locate possible additional localized zero modes.

We follow our method step by step. We hammer all the beads, each rotor once and each bead, associated with two degrees of freedom twice, in two different directions. We then compile our results to form the functions $\psi_{i}$, where *i* here indexes the different degrees of freedom. In the methods, we show the extent of each perturbation defining the mechanical molecules shown in Fig. 1*D*. Now that we have identified the bulk mechanical molecule (viz. the atomic-limit unit cell), we notice that it does not fully tesselate our metamaterial. Therefore, zero energy modes can in principle be found at the edges, and, or, the corners of the sample. To check whether they exist or not, we need to compute the average chiral polarization of each molecule both in the bulk and along the edges. To do so, we apply Eq. **6** to each mechanical molecule. We find that the chiral polarization differs in the bulk and in the edge regions, but the amplitude of the jump in Π is smaller than one lattice spacing. This local change in the chiral polarization does not correspond to a singularity in the continuum limit and therefore does not translate into any edge state. In stark contrast, we observe that the bottom and left edges feature two incompatible chiral polarization fields which feature a positive-divergence singularity at the bottom left corner. This observation implies that this corner must host a localized floppy mode. In order to check our prediction, we shake our metamaterial at increasing frequencies and find that the first vibrational mode is indeed localized at the bottom left corner as illustrated by the superimposed image sequence of Fig. 1*E*. The finite frequency of the vibration mode is due to the finite stiffness of the rotors’ design. In order to further confirm our predictions, we perform FEM simulations, compute the vibration spectrum of the full structure, and indeed find that the lowest energy mode is a floppy mode located at the bottom left corner (*SI Appendix*). We note that we have been extra cautious when measuring the chiral polarization of all mechanical molecules. An even faster protocol would have consisted in probing the chiral polarization of five molecules only: one in the bulk and one per edge. The experimental measurements reported in this section confirm the predictive power and the robustness of our method. We can detect experimentally all the topologically protected zero modes which live at the edge and corners of unknown mechanical networks without resorting to any theoretical model.

## Discussion

To close this article, we comment on the fundamental and practical implications of our findings. From a fundamental perspective, we note that the model studied in Fig. 4 is the paradigmatic example of a Higher-order Topological Insulator (HOTI) (40). HOTIs are usually characterized by elaborated invariants of tight-binding Hamiltonians such as the nested Wilson loops (40) or mirror-graded winding numbers (42), which rely on crystalline symmetries. However, in Fig. 4*D*, we show that even though the spatial average of Π is not a topological marker of HOTIs, its local measurement provides a very efficient tool to experimentally predict and locate their associated corner states. In mechanics, higher-order topological metamaterials are fundamentally chiral, therefore, as in electrostatics, the only two ingredients needed to detect localized zero-energy states (i.e., a local excess of chiral charges) are i) the incommensurability of the mechanical molecules with the global structure and ii) a local divergence of the chiral polarization field.

From a more practical perspective, reviving Kelvin’s wisdom in the context of topological mechanics has allowed us to provide a simple and practical method to identify chiral topological phases and detect their zero-energy modes. We stress in particular the relevance of our approach to probe the existence of states of self-stresses that would escape any form of inspection based on (linear) spectral measurements. At this stage, it is also worth recalling that our method is not specific to periodic lattices. The very concepts of mechanical molecules and chiral polarization exist in disordered networks as well and can be measured following the exact same procedures and formulas.

We conclude with two final remarks. We do not believe that Kelvin’s tenet should be opposed to metamaterial design. On the contrary, we expect that our experimental method could be effectively used as an effective optimization tool. We can think of tailoring mechanical metamaterials through genetic evolution or machine learning strategies over the chiral polarization, for example, to avoid, or promote, states of self-stress as local precursors of nonlinear response and failure (43).

Finally, beyond the specifics of mechanics, our method readily applies to any form of chiral matter: from photonic metamaterials where the response to light impulses has already been used to characterize Hamiltonian models (44–46), to acoustic (41, 47, 48), microwave (49), and electrical circuits (50–52).

## Materials and Methods

In the following, we provide the theoretical basis for the electrostatic analogy in mechanics, we work out in detail the one-dimensional case, and we further discuss the methodology proposed in the main text. In *SI Appendix*, we present a detailed validation for the one- and two-dimensional systems, using experiments, FEM simulations, and linear simulations. We also expand on the technical details regarding the data analysis, FEM simulations, and sample fabrication.

### 1. From Bead-and-Spring Networks to Chiral Hamiltonians.

In this section, we provide a brief introduction to the correspondence between the linear dynamics of bead-and-spring networks, and the quantum dynamics of a particle ruled by a chiral Hamiltonian. More specifically, we first define the linear chiral Hamiltonian H. Our derivation is an alternative to the original correspondence introduced by Kane and Lubensky (8).

Let us consider a collection of *N* beads of mass *m* and $N_{c}$ springs of stiffness *k* in *d* dimensions. We denote $u=(u_{1},u_{2},...,u_{N})$ the vector of displacements, and $e=(e_{1},e_{2},...,e_{N_{c}})$ the vector of elongations. u and e are geometrically related by the linear relation

[7] $$\begin{array}{c} e=Q^{\text{T}}u, \end{array}$$

where $Q^{\text{T}}$ is the so-called compatibility matrix. For a linear chain, $Q^{\text{T}}$ is nothing else but the discrete difference operator. Its transpose Q is the equilibrium matrix. It relates forces acting on the beads $f=(f_{1},f_{2},...,f_{N})$ to spring tensions $t=(t_{1},t_{2},...,t_{N_{c}})$ as:

[8] $$\begin{array}{c} f=Qt. \end{array}$$

Newton’s equations, together with Hooke’s law t=ke, define the dynamics of the beads in the linear response regime:

[9] $$\begin{array}{c} \ddot{u}=-\frac{k}{m}QQ^{\text{T}}u. \end{array}$$

In the simplest case of a linear collection of beads connected by springs, $QQ^{\text{T}}$ reduces to the discrete Laplace operator. It is worth noting that although less standard, the dynamics of the mechanical structure can be expressed as a function of the elongation variables simply by using the compatibility relation:

[10] $$\begin{array}{c} \ddot{e}=-\frac{k}{m}Q^{\text{T}}Qe. \end{array}$$

These two equations can be combined into a single differential equation in terms of the joint vector of displacements and elongations $|\Psi \rangle =(u,e)$:

[11] $$\begin{array}{c} \left(\partial_{t}^{2}-H^{2}\right)|\Psi \rangle =0, \end{array}$$

where $H=\left(\begin{array}{cc} 0 & Q\\ Q^{\text{T}} & 0 \end{array}\right)$, and units are chosen such that k/m=1 for simplicity. The operator on the left-hand side can be recast as the product of two commuting operators $\left(\partial_{t}^{2}-H^{2}\right)=-\left(i\partial_{t}-H\right)\left(i\partial_{t}+H\right)$; thus, Eq. **11** is equivalent to the Schrödinger equation:

[12] $$\begin{array}{c} i\partial_{t}|\Psi \rangle =H|\Psi \rangle . \end{array}$$

#### A. Chiral symmetry and electrostatic analogy.

It is crucial to note the chiral nature of the Hamiltonian, H, defined by the anticommutation relation $\left\{C,H\right\}=0$, where the unitary chiral operator C is represented by the diagonal matrix $C_{\mathrm{ij}}=\delta_{\mathrm{ij}}$ for i≤dN and $C_{\mathrm{ij}}=-\delta_{\mathrm{ij}}$ otherwise. We can readily state some straightforward consequences of this fundamental symmetry. First, chiral symmetry implies that for each eigenstate $|\Psi_{E}\rangle$ of finite energy *E*, there exists a chiral partner $|{\text{Ψ}}_{-E}\rangle =C|{\text{Ψ}}_{E}\rangle$ of opposite energy −E. The spectrum of H is thus symmetric. Taking the opposite sign of H in Eq. **12** therefore describes the same physics. However, zero energy modes do not come by pairs of eigenstates and are eigenstates of C with eigenvalues +1 (resp. −1) for floppy modes (resp. self-stress states). Second, by virtue of the Maxwell–Calladine index theorem, isostatic systems correspond to those having as many constraints as displacement degrees of freedom, i.e., a null total chiral charge defined as tr(C)=0 (32).

Finally, the structure of C begs for an electrostatic analogy where the displacement degrees of freedom plays the role of positive charges while the elongations play the role of negative charges, and positive charges only interact with negative charges. At this stage the analogy is merely superficial, we give it some substance in the following sections.

### 2. Theoretical Basis for the Model-Free Characterization of Mechanical Structures.

#### A. Wannier states and mechanical molecules.

We first provide a theoretical benchmark to validate the model-free measurements discussed in the main text. To do so, we recall some concepts thoroughly discussed in ref. 32. Our starting point is the linear chiral Hamiltonian H defined in Section 1 and an ensemble of Wannier states associated with the negative spectrum. While Wannier states are commonly defined as localized states built out of eigenstates of a single energy band, their definition can be extended to a set of bands. In the present case, we focus on multiband Wannier states associated with the negative energy spectrum, serving as a spatially localized basis of H. This set of Wannier functions is not unique. Many variations exist, depending on the degree of localization or their complex phases. However, in the context of electrons in crystals, maximally localized Wannier functions provide a good representation of atomic orbitals (37). By extension, in our experiments we expect the Wannier functions of H to define the support of “mechanical molecules,” i.e., the pair of degrees of deformation and constraints that are dominantly coupled, similarly to an atomic orbital for electrons.

#### B. How to compute the Wannier functions.

For 2D metamaterials, we define the Wannier functions as the set of functions spanning the negative energy space and minimizing the spreading functional:

[13] $$\begin{array}{c} \Omega \left[\left\{W_{i}\right\}\right]=\frac{1}{N}\sum_{j}^{N}\left(\langle W_{j}|R^{2}\left|W_{j}\right\rangle -{\left|\langle W_{j}|R|W_{j}\rangle \right|}^{2}\right), \end{array}$$

where R is the position operator. Alternative methods such as the matrix pencil method lead to sets of comparable localization (32).

For one-dimensional systems, a maximally localized basis of Wannier functions corresponds to the eigenstates of the projected position operator $PR_{x}P$, where $P=\underset{E<0}{\sum }|{\text{Ψ}}_{E}\rangle \langle {\text{Ψ}}_{E}|$. Obviously, in a periodic lattice distinct Wannier functions are related by unit-cell translations, $\langle x\rangle W_{i}=\langle x-ja\rangle W_{i-j}$. Note that by definition, the Wannier functions are normalized: $\langle W_{i}\rangle =1$.

#### C. Charge, location, and polarization of mechanical molecules.

We use the electrostatic analogy introduced in Section A to define the charge, the location, and the polarization of mechanical molecules.

##### Chiral charge.

We introduce the concept of chiral charge as:

[14] $$\begin{array}{c} c_{W_{i}}=\langle W_{i}\left|C\right|W_{i}\rangle . \end{array}$$

It represents the difference between the average number of deformation degrees of freedom (+1 charges) and constraints (−1 charges) within a mechanical molecule defined by the support of a Wannier function.

##### Chiral molecules.

Similarly, we naturally define the center of mass of the Wannier function as:

[15] $$\begin{array}{c} r_{W_{i}}=\langle W_{i}\left|R\right|W_{i}\rangle . \end{array}$$

In a periodic lattice, the $r_{W_{i}}$ are deduced from one another by unit-cell translations.

##### Chiral polarization.

Finally, as in conventional charged systems, we can define the chiral polarization of our mechanical molecules as:

[16] $$\begin{array}{c} \Pi_{W_{i}}=2\left(\langle W_{i}\left|CR\right|W_{i}\rangle -c_{W_{i}}r_{W_{i}}\right). \end{array}$$

#### D. A tutorial example: theoretical chiral polarization field in the periodic mechanical SSH chain.

**Fig. 5.**
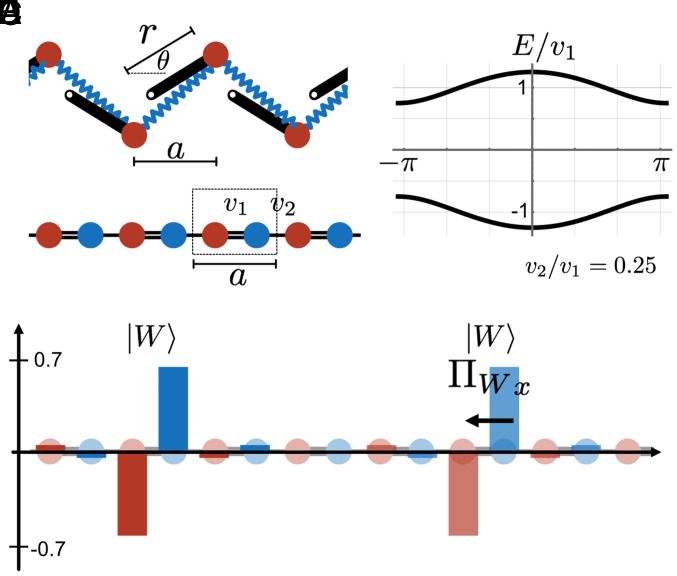
Periodic SSH model of the mechanical chain. (*A*) The equilibrium configuration is defined by the rotor length *r*, the unit-cell spacing *a*, and the equilibrium angle θ. (*B*) Chiral one-dimensional representation of the mechanical chain. Beads and springs are symbolized by red and blue circles, respectively. Perturbations around the equilibrium are described by the linear Hamiltonian H known as the SSH model. Here, $v_{2}<v_{1}$. (*C*) Spectrum for a periodic chain using the experimental values r=25.4 mm, a=60 mm, and θ=π/4. (*D*) Wannier functions are localized on the strongly linked sites (in this case $v_{1}$) and are translations of one another. (*E*) The chiral polarization is translationally invariant and connects the strongly connected sites. Here we represent only the horizontal component.

The mechanical chain of rotors under periodic boundary conditions is described by the SSH model (8); see Fig. 5 *A* and *B*. Exploiting the periodicity of the system, the Bloch Hamiltonian is described in momentum space as:

[17] $$\begin{array}{c} H_{\text{SSH}}\left(k\right)=\left(\begin{array}{cc} 0 & v_{1}+v_{2}e^{\mathrm{ika}}\\ v_{1}+v_{2}e^{-ika} & 0 \end{array}\right), \end{array}$$

where the parameters $v_{1}$ and $v_{2}$ depend on the specific geometry of the metamaterial. They depend on the length of the rotor *r*, the equilibrium angle θ, and the unit-cell length *a*:

[18] $$\begin{array}{cc} v_{1} & =r\sin\theta \frac{a+2r\sin\theta }{\sqrt{a^{2}+4r^{2}\sin^{2}\theta }}, \end{array}$$

[19] $$\begin{array}{cc} v_{2} & =r\sin\theta \frac{a-2r\sin\theta }{\sqrt{a^{2}+4r^{2}\sin^{2}\theta }}. \end{array}$$

The equilibrium position operator R is a diagonal matrix whose entries are the vectors of equilibrium positions of beads and springs: $R=\text{diag}\left(r_{{\text{bead}}_{1}},r_{{\text{bead}}_{2}},...,r_{{\text{bead}}_{N}},r_{{\text{spring}}_{1}},...,r_{{\text{spring}}_{N_{c}}}\right)$, where the equilibrium positions are given by:

[20] $$\begin{array}{cc} r_{{\text{bead}}_{j}} & =(ja+r\cos\theta ,{\left(-1\right)}^{j}r\sin\theta ), \end{array}$$

[21] $$\begin{array}{cc} r_{{\text{spring}}_{j}} & =((j+1/2)a+r\cos\theta ,0). \end{array}$$

Since the problem in question is essentially one-dimensional, we are only interested in the horizontal component of the position operator. Its Fourier transform takes the simple form:

[22] $$\begin{array}{c} R_{x}\left(k\right)=\left(\begin{array}{cc} r\cos\theta +i\partial_{k} & 0\\ 0 & a/2+r\cos\theta +i\partial_{k} \end{array}\right). \end{array}$$

In an infinite or a periodic chain, the chiral charge linked to a single Wannier function also corresponds to the average chiral charge tr(C) of the whole system:

[23] $$\begin{array}{c} c_{W_{i}}=\langle W_{i}\left|C\right|W_{i}\rangle =\frac{\text{tr}\left(CP\right)}{\text{tr}(P)}=\frac{\text{tr}(C)}{2\text{tr}(P)}=0, \end{array}$$

where the last equality follows from the isostaticity of the network, the number of degrees of freedom is equal to the number of springs($N=N_{c}$). The center of charges in a periodic chain, the $r_{W_{i}}$, are deduced by one another by a unit-cell translation (Fig. 5*D*).

Finally, we can define the chiral polarization of our mechanical molecules using Eq. **16**. Noting that the chiral charge is zero in the isostatic chain, we find that $\left\langle W_{i}|CR|W_{i}\right\rangle$ is origin independent. In addition, as the Wannier functions are translations of one another, we can simplify Eq. **16** as:

[24] $$\begin{array}{c} {\left(\Pi_{W_{i}}\right)}_{x}=2\langle W_{i}|CR_{x}\left|W_{i}\right\rangle =2\frac{\text{tr}(CR_{x}P)}{\text{tr}(P)}=\text{sign}\left(v_{2}-v_{1}\right)\frac{a}{2}. \end{array}$$

The above expression can be recast into the compact form $\Pi_{x}=wa-a/2$, with $w=\frac{1}{2}\left[\text{sign}\left(v_{2}-v_{1}\right)+1\right]$. In ref. 32, we showed that the integer *w* is nothing else but the conventional topological index of chiral topological phases, i.e., the winding number of H. Distinct topological phases are therefore characterized by opposite chiral polarizations (Fig. 5*E*).

#### E. Chiral polarization field from Wannier functions in finite mechanical chains.

**Fig. 6.**
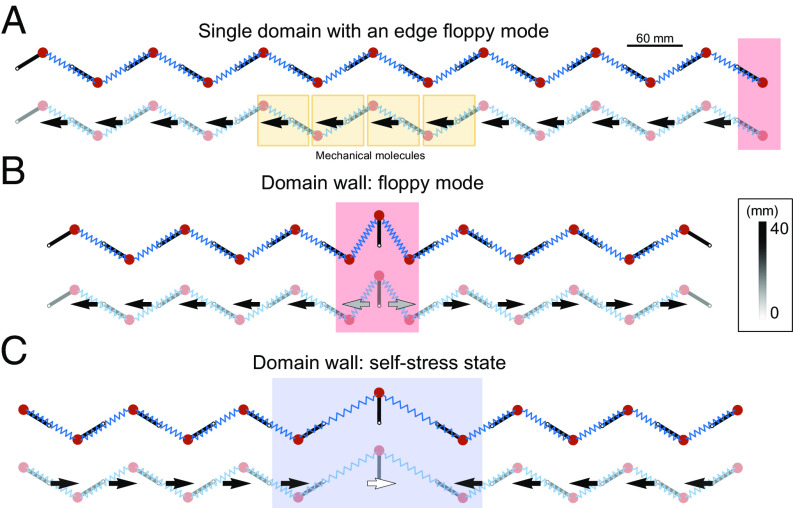
Theoretical chiral polarization field for the three examples shown in the main text: a single domain (*A*), two domains connected by a floppy mode (*B*), and two domains connected by a self-stress state (*C*). Each case shows the finite chain (Top) and chiral polarization field (Bottom). The vector field is represented by constant-length arrows with magnitude indicated by the black-and-white color bar. In all cases, we use the experimental parameters: rotor length 25.4 mm, unit-cell length 60 mm, and equilibrium angle |θ|=π/4.

Fig. 6 illustrates the theoretical chiral polarization computed from the numerical values of the Wannier functions in three types of mechanical chains. Fig. 6*A* shows a homogeneous system hosting a floppy mode at one end, representing the experimental chain of Fig. 2*A*. We illustrate the support of the chiral molecules, their center, the localized floppy mode at the right edge, and the chiral polarization field. In Fig. 6 *B* and *C*, we show two heterogeneous chains assembled from two distinct topological phases characterized by opposite chiral polarizations, representing the systems of Fig. 3 *A* and *E*. We emphasize that, as in electrostatics, a discontinuity of the chiral polarization field implies the existence of an isolated chiral charge at the junction between the two incompatible topological phases. This observation is known as the bulk-boundary correspondence and was thoroughly discussed in ref. 32. More precisely, a discontinuity of the chiral polarization field of the order of one unit-cell length: $|\Pi_{x}^{\text{left}}-\Pi_{x}^{\text{right}}|\geq a$ distinguishes two distinct topological phases. Floppy modes and self-stress states are identified by source and sink-like discontinuities respectively (32), Fig. 6 *B* and *C*.

### 3. Methodology.

From the previous section, we learned that the chiral polarization field is determined by the ensemble of Wannier functions, which uniquely define the system’s molecules and their polarization. While it is impractical to determine these functions experimentally, we can instead use the dynamics of localized mechanical perturbations to bypass this need.

#### A. The shortcomings of Wannier functions.

In a nutshell, knowing the low-energy eigenstates we can compute the local functions defining the chiral molecules and, ultimately, the chiral polarization field. In principle, the low-energy eigenstates could be experimentally measured by vibrating the sample across varying frequencies and identifying the modes where local fluctuations peak. While appealing in theory, this approach is doomed to fail. Although the eigenenergies are easily accessible, see SI Appendix of ref. 41, determining a set of low-energy chiral eigenstates is out of reach of any realistic experiment. Differences arise from measurement precision, inherent experimental noise, or mechanical nonlinearities not accounted for by H. Experimentally derived eigenmodes, as a result, are not exclusively chiral, encompassing projections on both $P=P_{E<0}$ and $P_{E>0}=I-P$. This inevitable feature makes it impossible to define chiral molecules and their polarization from experimentally measured modes. In the next sections, we show how to circumvent this fundamental limitation, and, more precisely, how to use local mechanical excitations as effective proxies for Wannier functions.

#### B. Dynamics of the Wannier function in the mechanical chain.

To gain some insight, we first investigate the Wannier functions’ dynamics and their impact on chiral charges and polarizations. The unitary evolution operator is given by $U_{t}=e^{-itH}$. Starting from a Wannier state $|W_{i}\rangle$, the wave function at time *t* is given by $|W_{i}(t)\rangle =U_{t}|W_{i}\rangle$.

Using Eq. **14** we find:

[25] $$\begin{array}{cc} c_{W_{i}\left(t\right)} & =\langle W_{i}|U_{-t}CU_{t}\left|W_{i}\right\rangle =\frac{\text{tr}(U_{-t}CU_{t}P)}{\text{tr}(P)}\\ & =\frac{\text{tr}(U_{-t}CPU_{t})}{\text{tr}(P)}=\frac{\text{tr}(CP)}{\text{tr}(P)}=0, \end{array}$$

where we use the periodicity of $|W_{i}\rangle$ to simplify the second equality. The third equality simplifies thanks to the commutation relation $[P,U^{t}]=0$, and the fourth equality follows from the cyclicity of the trace operation. Ultimately, we find that the chiral charge is time-independent and equal to zero in the isostatic SSH chain.

The same reasoning applies to the chiral polarization itself. The chiral polarization is time independent: ${\left(\Pi_{W_{i}}\right)}_{x}\left(t\right)=\text{sign}\left(v_{2}-v_{1}\right)\frac{a}{2}$.

#### C. Numerical validation in the finite mechanical chain.

We now show that the long-time average of the chiral charge, position, and polarization does not fundamentally require initializing the dynamics with a pure Wannier state. We numerically show that any localized mechanical perturbation can identify the Wannier states’ support, center, and chiral polarization.

**Fig. 7.**
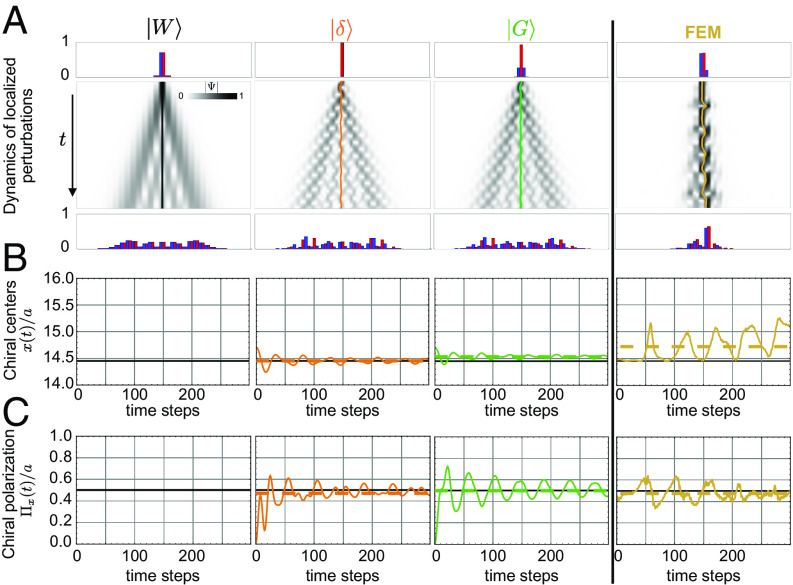
Local dynamics as proxies of the Wannier functions: Numerical validation in the one-dimensional model. (*A*) Evolution of four distinct localized perturbations in a 31-rotor chain, all centered in the middle of the finite system. From Left to Right: Linear evolution of a Wannier function $|W\rangle$, a fully localized function on one degree of freedom $|\delta \rangle$, and a Gaussian function *G*, and a finite element simulation of a single-node displacement. In all linear cases, the initial state spreads at a rate fixed by the stiffness and the mass of the beads $\;\sqrt{k/m}=1$. At each time step, we compute the chiral center (*B*) and the chiral polarization (*C*), normalized by the unit-cell length *a*. Only the Wannier maintains a constant time signal, while others oscillate around the expected value, with the chiral center and polarization defined as the time average of the signal (dashed line). Beyond the linear Hamiltonian, FEM simulations display the same behavior. In all cases, we display 300 time-steps. For the linear simulations, the time step is dt=0.1 and a=1 (dimensionless units). For the FEM simulation, dt=0.03 ms and a=60 mm.

As a benchmark comparison, we first detail the one-dimensional mechanical chain of rotors. Fig. 7 compares the evolution of Wannier functions with two localized wave functions: one localized on a single degree of freedom and a Gaussian function of a width comparable with the unit-cell length *a*. All perturbations spread at a speed defined by $v_{1}/v_{2}$, with wave functions taking longer to spread over weaker interaction links. Fig. 7*D* reveals a consistent time average center of the mechanical molecule ${\left\langle x\left(t\right)\right\rangle }_{t}$ across functions. This consistency also applies to the chiral polarization, which oscillates around the theoretical value (Fig. 7*E*).

We finally compare our linear model to the full FEM resolution of the mechanical problem (*Rightmost* panels of Fig. 7). We study the dynamics of a structure identical to our 3D-printed material in response to the displacement of a single rotor at t=0. Both the molecule’s position and chiral polarization fluctuate around the theoretical values from our simple linear model. This underscores our method’s reliability in gauging chiral molecule centers and polarizations using local poking experiments.

#### D. Discussion: linear numerics vs. experiments.

In the previous section, we show how the linear dynamics provides an excellent proxy for the Wannier functions in mechanical networks. In reality, however, the dynamics due to local perturbations are subject to two major effects noncaptured by the previous models: damping and nonlinear effects.

Damping breaks the chiral formulation presented in Section 1. The addition of velocity-dependent forces forbids the chiral Hamiltonian decomposition of Eq. **12**. Nonetheless, the intrinsic difference between the beads and springs does not depend on any possible dissipation process. This is where mechanics differs from other chiral platforms, such as electronics or photonics: The sublattice symmetry is intrinsic and not limited to any linear theory. The simple and natural distinction between beads and springs is enough to accurately compute the chiral polarization field.

From an experimental point of view, the inclusion of damping is even desirable as it filters out the weakest interactions, giving even clearer signals of the strongly coupled degrees of freedom and constraints.

Finally, it is worth pointing out that in all the model-based descriptions, an a priori knowledge of the degrees of freedom is required. For example, the degree of freedom of a rotor is the angle variable θ and not the displacement vector u. The model-free characterization shown in the main text does not rely on this information. All the data are treated in the most generic form: displacements and elongations. In *SI Appendix*, we detail the data reconstruction from experiments and finite element (FE) simulations, without resorting to any linear modeling.

## Supplementary Material

Appendix 01 (PDF)

Movie S1. Vertical vibration of the presented 2D metamaterial. a corner floppy mode is observed as the first mode at 88 Hz.

## Data Availability

Code data have been deposited in Zenodo (model-free-topological-mechanics) https://doi.org/10.5281/zenodo.7799768 (53).

## Footnotes

^*^Not to be confused with the finite energy chiral symmetry of the dynamical matrix, present, for instance, when dimerizing the spring constants of a one-dimensional array of beads.
