# Supplementary material for: Model-free characterization of topological edge and corner states in mechanical networks
Source: Proc Natl Acad Sci U S A. 2024 Jan 17;121(4):e2305287121. doi: 10.1073/pnas.2305287121 (PMC10823249; doi:10.1073/pnas.2305287121)
Supplement: Supplementary file 1 — Appendix 01 (PDF) [file pnas.2305287121.sapp.pdf]

Supporting Information for

Model-free characterization of topological edge and corner states in mechanical networks

Marcelo Guzmán, Xiaofei Guo, David Carpentier, Corentin Coulais, Denis Bartolo

Marcelo Guzmán.
E-mail: mguzmanj@sas.upenn.edu

This PDF file includes:

Supporting text
Figs. S1 to S22
Legend for Movie S1
SI References

Other supporting materials for this manuscript include the following:

Movie S1

Supporting Information Text

1. Validation: Two-dimensional mechanical system

A. Chiral polarization field from Wannier functions in 2D mechanical metamaterial. Here we provide a benchmark comparison for the experimental measurements of the chiral polarization field shown in Fig.1 of the main text.

Figure S1 depicts the linear modelization of the system as a collection of beads and springs. The unit cell contains four beads and six springs: the metamaterial is hyperstatic. From the local connectivity we find the compatibility matrix and its Fourier transform, Fig. S1c, from which we construct the Hamiltonian $\mathcal{H}$.

In momentum space, the compatibility matrix is rectangular as there is one non-compensated constraint in each unit cell. The corresponding Hamiltonian, therefore, enjoys a zero-energy flat band, Fig. S1d. The system is hyperstatic, and the number of self-stress states is extensive in the bulk.

In addition to the bulk states of self-stress, a finite system of 3×4 unit cells, Fig. S2a, hosts a localized floppy mode in the bottom-left corner, Fig. S2b. In contrast to the one dimensional case, for which each unit-cell mapped exactly to one Wannier function, here the mapping is no longer bijective: each unit cell contains several Wannier functions. This is explicitly shown by the distribution of Wannier centers, Fig. S2c. Each Wannier function defines its own polarization, Fig. S2d, leading to a discontinuous field at the sub unit-cell scale. The spatial distribution of the centers hints towards a coarse-graining procedure: we add the chiral polarization from Wannier functions whose centers are separated by less that one lattice spacing. The coarse grained field is shown in fig. S2e, indicating a discontinuity on the bottom-left corner, at the scale of the unit-cell size, where the zero zero-energy mode is located.

We can quantify this discontinuity through the discrete divergence of the chiral polarization field, Δ. We define this quantity in the corner as $\Delta = |(\mathbf{\Pi}_{\rm top} - \mathbf{\Pi}_{\rm corner}) \cdot \hat{y} + (\mathbf{\Pi}_{\rm right} - \mathbf{\Pi}_{\rm corner}) \cdot \hat{x}|$, where top and right refer to the molecules next to the corner. Since no Wannier function is defined in the corner, we have $\mathbf{\Pi}_{\rm corner} = \mathbf{0}$. From the coarse-grained field we obtain that $\Delta = 207.9\text{mm} > 120\text{mm}$.

B. Numerical validation in 2D mechanical system. Following the same benchmark procedure as in our first 1D example, we compare the full chiral polarization field obtained from three types of localized initial conditions: Wannier functions, perturbations localized on one bead, and Gaussian functions, Fig. S3a. We compute the time evolution of the three initial conditions using the same Hamiltonian $\mathcal{H}$ for a finite system of 3×4 unit cells, and choose units such that $k/m = 1$.

Unlike the one-dimensional case, here the Wannier functions do not lead to time-independent chiral moments. Instead, they evolve in time. The same observation holds for the other two initial conditions. The centers explore confined areas, the beads and springs enclosed in these regions define the mechanical molecules. To identify the beads and springs within a mechanical molecule in 2D and 3D metamaterials, we use the Mahalanobis distance associated to the distribution $\Psi(t)$ and construct polygons spanning the strongly related sites. For details, see section 2B.

While the exact shape of the polygons depends on the specifics of the initial perturbation, they all reveal a very similar underlying structure: the mechanical molecules. We can now compute the associated coarse-grained chiral polarization field averaged over time, Fig. S3g, h, and i. In all three cases, we find chiral polarization fields with the same gross features and in particular sharing the same discontinuity at the bottom-left corner: the signature of a topologically protected zero corner mode, Fig. S2e.

As before, we can compute the discontinuity of the coarse-grained chiral polarization field, in the corner highlighted by the dashed square in the bottom row of Fig. S3. For the Wannier case, $\mathbf{\Pi}_{\rm corner} = \mathbf{0}$. For the three cases explored, namely Wannier, fully localized, and Gaussian, we obtain respectively $\Delta = 232.9\text{mm},\ 54.4\text{mm},\ 93.3\text{mm}$, larger (or at least of the order) of the corner region/smallest molecule $\sim 60\text{mm}$.

2. Definition of the wave functions and mechanical molecules from raw experimental and numerical data

In this section we detail the data analysis carried out from the raw data of displacements and elongations in both the one- and two-dimensional mechanical systems discussed in the main text.

A. Mechanical chain. Both the simulations and experiments give us access to the planar displacements of the beads, $\boldsymbol{u}_i(t) = (u_{i,x}(t), u_{i,y}(t))$, with i indexing each bead. If two beads, say i and j, are connected by a spring, its elongation is computed as

$$e_{i,j}(t) = |(\boldsymbol{r}_j^{\rm eq} + \boldsymbol{u}_j(t)) - (\boldsymbol{r}_i^{\rm eq} + \boldsymbol{u}_i(t))| - |\boldsymbol{r}_j^{\rm eq} - \boldsymbol{r}_i^{\rm eq}|, \quad [1]$$

with $\boldsymbol{r}_i^{\rm eq}$ the equilibrium position of the i-th bead.

Fig. S4 illustrates the raw displacements and elongations of all the nodes for the one-dimensional mechanical chain of rotors. From a perturbation applied to the bead i, we directly compute the wave functions Ψ_i, its norm, its center $\mathbf{r}_i(t)$, the chiral charge, and the chiral polarization, Fig. S10. In reality, the metamaterial dissipates mechanical energy. As a result the perturbations do not freely propagate but are damped in a finite time. Beyond the damping time, the signal corresponds to noise, leading to spurious measurements in terms of the chiral moments. To filter out this unwanted noise we restrain our measurements to the time intervals in which the norm is higher than a threshold ϵ. For our experiments, we set $\epsilon_{\rm exp} = 0.3$.

Once the data are filtered, we compute the time average center and chiral polarization. We show in the SI that changing the extent of the time averaging window results in insignificant variations of our observables.

a.

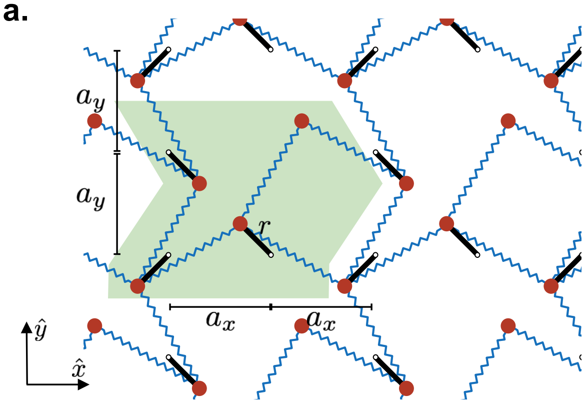

b.

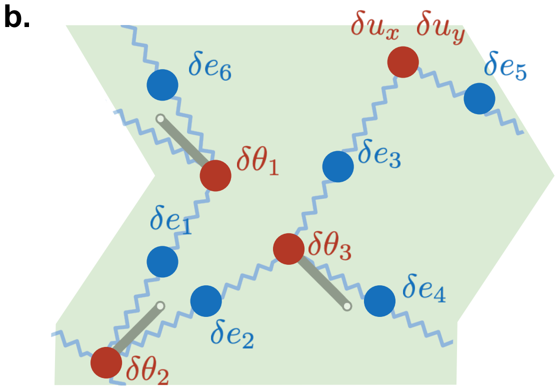

c.

$$\mathcal{Q}^\dagger(k_x,k_y) = \begin{array}{c|ccccc} & \delta\theta_1 & \delta\theta_2 & \delta\theta_3 & \delta u_x & \delta u_y \\ \hline \delta e_1 & \frac{\sqrt{2}a_y+2r}{2\sqrt{a_y^2+2r^2}} & \frac{\sqrt{2}a_y-2r}{2\sqrt{a_y^2+2r^2}} & 0 & 0 & 0 \\ \delta e_2 & 0 & \frac{2r-\sqrt{2}a_x}{2\sqrt{a_x^2+2r^2}} & \frac{-\sqrt{2}a_x-2r}{2\sqrt{a_x^2+2r^2}} & 0 & 0 \\ \delta e_3 & 0 & 0 & \frac{\sqrt{2}a_y+2r}{2\sqrt{a_y^2+2r^2}} & \frac{\sqrt{2}r}{\sqrt{a_y^2+2r^2}} & \frac{a_y}{\sqrt{a_y^2+2r^2}} \\ \delta e_4 & 0 & \frac{e^{-2ia_xk_x}\left(a_x+\sqrt{2}r\right)}{\sqrt{2}\sqrt{a_x^2+2r^2}} & \frac{a_x-\sqrt{2}r}{\sqrt{2}\sqrt{a_x^2+2r^2}} & 0 & 0 \\ \delta e_5 & \frac{e^{-2ia_xk_x}\left(\sqrt{2}a_x-2r\right)}{2\sqrt{a_x^2+2r^2}} & 0 & 0 & -\frac{a_x}{\sqrt{a_x^2+2r^2}} & \frac{\sqrt{2}r}{\sqrt{a_x^2+2r^2}} \\ \delta e_6 & -\frac{a_y-\sqrt{2}r}{\sqrt{2}\sqrt{a_y^2+2r^2}} & \frac{e^{-2ia_yk_y}\left(-a_y-\sqrt{2}r\right)}{\sqrt{2}\sqrt{a_y^2+2r^2}} & 0 & 0 & 0 \end{array}$$

d.

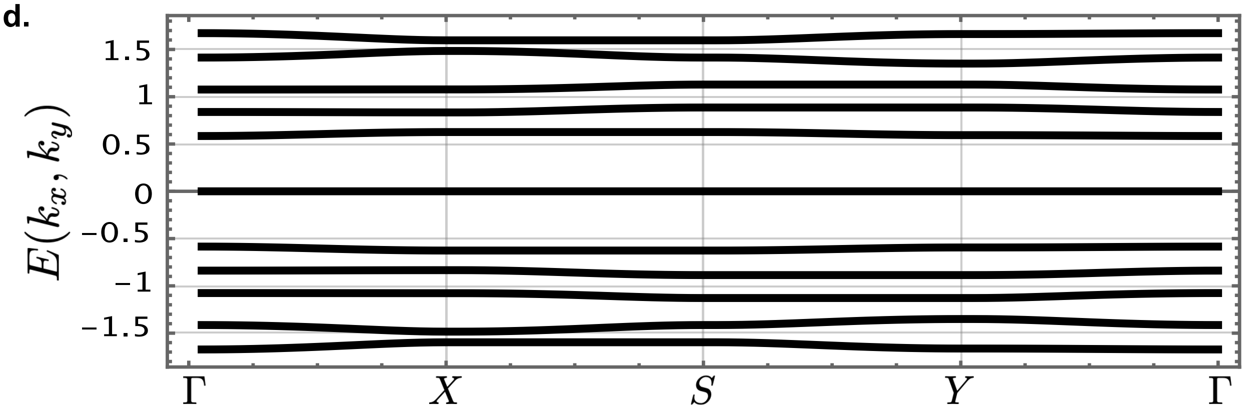

Fig. S1. Linear model of the 2D mechanical metamaterial. a. Periodic collection of bead-and-springs modelling the 2D metamaterial shown in the main text. The unit-cell is highlighted in green. All the equilibrium angles are assumed to be $\pm\pi/4$ with respect to the x axis. **b.** Displacements and elongations in the chiral representation inside the unit cell. **c.** Compatibility matrix in momentum space, according to the variables indicated in **b**. **d.** Eigenvalues of the Bloch Hamiltonian along the high-symmetry points of the square Brillouin zone. The spectrum is symmetric due to the chiral symmetry. The zero-energy flat band reflects the hyperstaticity of the system.

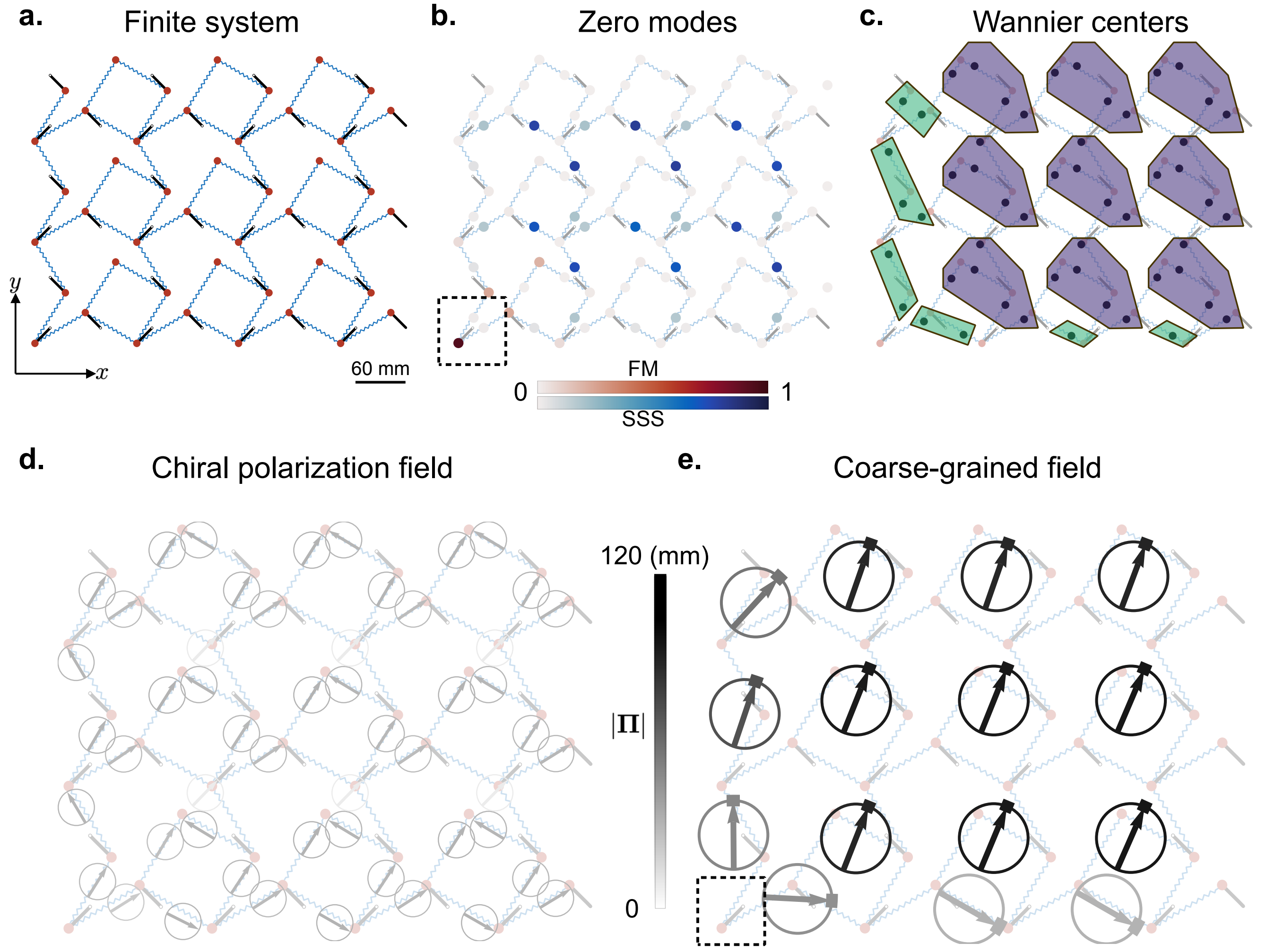

Fig. S2. Model-based chiral polarization field of the 2D mechanical metamaterial. a. Finite system of 3×4 unit cells, as used in the experimental setup. **b.** Zero-energy mode distribution. The color indicates the weight on degrees of freedom (red) and constraints (blue). The bulk presents an extensive number of self-stress states, whereas only one floppy mode is localized in the bottom-left corner (dashed square). **c.** Distribution of Wannier centers (black dots). Beginning from the bulk, we group the centers by 1) respecting the periodicity of the sample (i.e. groups of 5 Wannier centers) and 2) minimizing the spreading among them. These bulk molecules are highlighted in violet. The remaining centers are grouped into small edge molecules (green) according to their proximity. **d.** Chiral polarization field issued from all the Wannier functions. For readability, we represent the vector field with arrows of fixed length enclosed by a circle. The magnitude of the vectors is depicted by the gray scale and the angular deviations are represented by the thicker portion of the circle. **e.** Coarse-grained chiral polarization field. In the bottom-left corner (dashed square) the discrete divergence is larger than the lattice spacing: $\Delta = 207.9$mm > 120mm. The discontinuity signals the presence of the localized floppy mode.

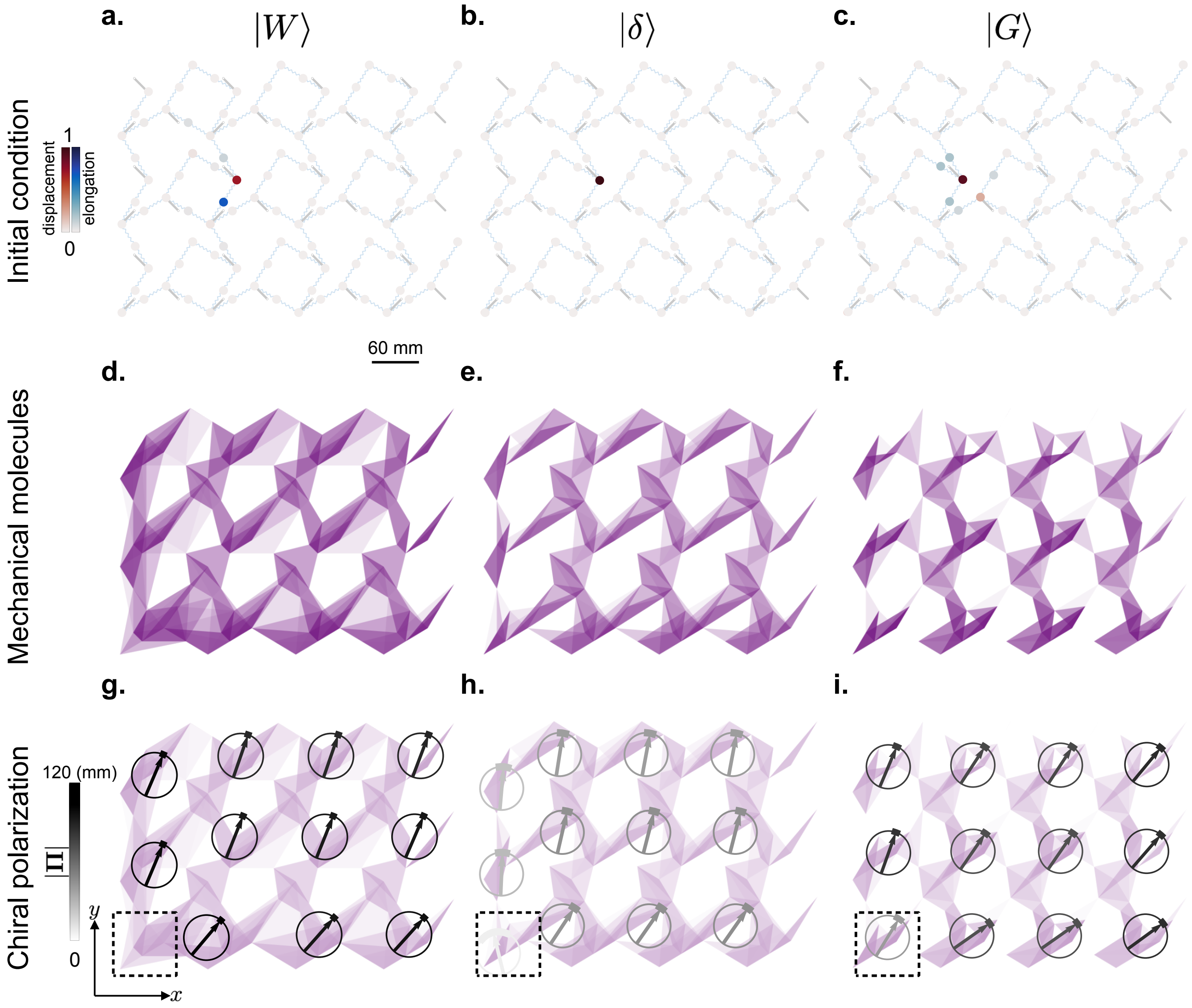

Fig. S3. Local dynamics as proxies of the Wannier functions: Numerical validation in the two-dimensional model. Comparison of the chiral molecules and chiral polarization field issued from the dynamics of Wannier $|W\rangle$, fully localized $|\delta\rangle$, and Gaussian functions $|G\rangle$ over the same system. One example of each initial condition is depicted in **a**, **b**, and **c**. The fully localized functions are taken only over the beads (degrees of freedom). The Gaussians have a standard deviation of $\sigma = 20$mm. **d, e, f.** Extent of the perturbations represented by the polygons for which the Mahalanobis distance d is smaller than 1.8. This is done for every time step with polygons of light opacity. Thus, the darker regions are the most persistent. **g, h, i.** Coarse-grained chiral polarization field for each case. The color of the arrow is linearly related to its magnitude. By definition, the Wannier functions do not span the regions of zero-energy mode, explaining the lack of polarization in the bottom-left corner. The discontinuity in the field matches the qualitative picture obtained from the spectral computation of fig. S2e.

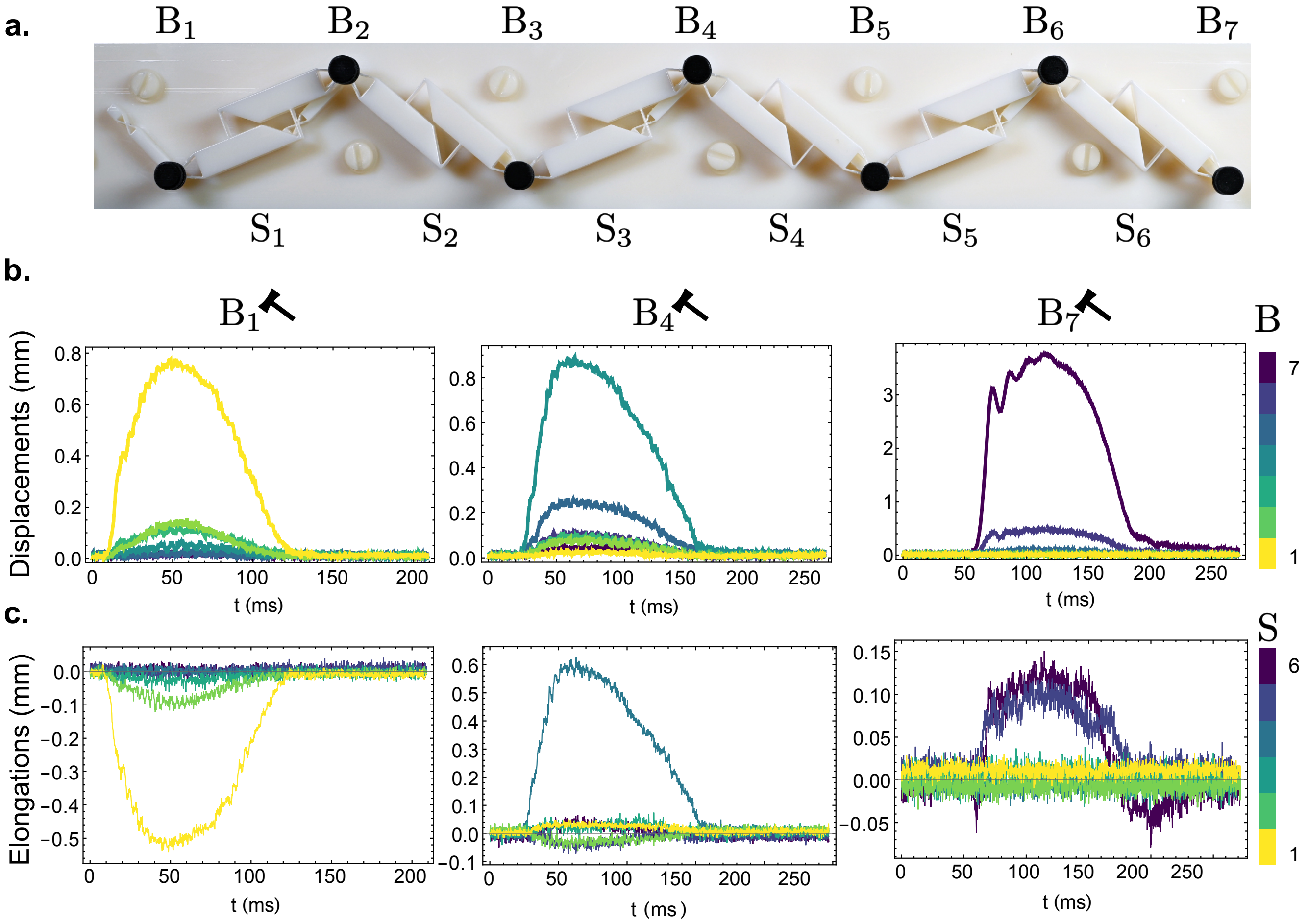

Fig. S4. Raw displacements and elongations from local perturbations in the mechanical chain (Experiments). a. Mechanical chain with labels for beads B_i and springs S_i. The floppy mode is hosted on bead B_7. A local excitation leads to a displacement (**b**) and an elongation (**c**) response. Here we illustrate three distinct perturbations on beads B_1, B_4, and B_7 (from left to right).

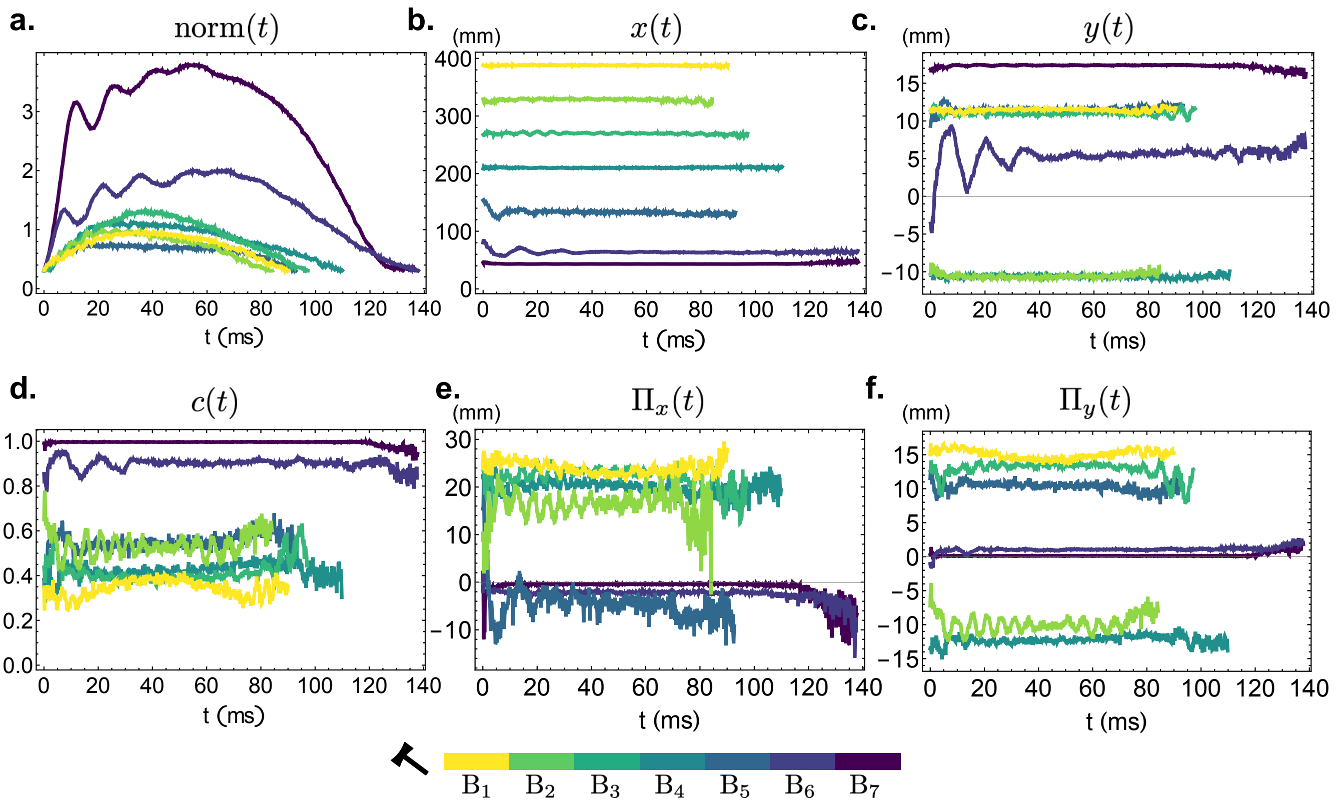

Fig. S5. Moments from local perturbations in the mechanical chain (Experiments). Norm (**a**), positions (**b** and **c**), chiral charge (**d**) and chiral polarization components (**e** and **f**), for all the different perturbations in the mechanical chain (color).

The mechanical molecules are directly extracted from the $N\%$ confidence interval of the distribution $\Psi(t)$. The value of N depends on the nature of the experiment/simulation. In our linear simulations there is no dissipation process. The perturbations hence unboundedly spreads across the whole system, as shown in Fig. 7. In this case, small values of N (below 30%) are well suited to capture the local asymmetric response. This educated choice is determined by the duration of the simulation. We typically set the value of N so that the confidence interval of Ψ does not exceed a distance much larger that the lattice spacing. We would otherwise define aggregates of the elementary molecules

In the experiments, on the other hand, dissipation quickly dampens the response and high values of confidence (95%) accurately captures the molecules, see Fig.2f.

The appropriate value of N depends not only on the friction but also on the duration of the experiment, as well as the inherent speed of sound of the material. In general, a good measurement strategy is to begin with small confidence values and slowly increase it until the first patterns of strongly correlated atoms separated by less than one lattice spacing emerge.

B. 2D mechanical system. In the two dimensional system, the same data acquisition protocol applies (see SI for the raw measurements).

The detection of the molecules, however, relies on a generalization of the concept of confidence interval. In this work we chose the Mahalanobis distance (1) $d(\boldsymbol{q}, \Psi(t))$ corresponding to how many standard deviations away the point $\boldsymbol{q}$ is from the mean of the distribution $\Psi(t)$, $\boldsymbol{r}(t)$. It is mathematically defined as

$$d(\boldsymbol{q}, \Psi(t)) = \sqrt{(\boldsymbol{q} - \boldsymbol{r}(t))S^{-1}(\boldsymbol{q} - \boldsymbol{r}(t))}, \qquad [2]$$

with S being the covariance matrix. We can then define the spread of the two-dimensional distribution $\Psi(t)$ as the region in space containing all the points $\boldsymbol{q}$ for which $d(\boldsymbol{q}, \Psi(t))$ is smaller than a threshold δ. This threshold is akin to the confidence value in 1D. Similarly to the 1D case, a good general strategy is to begin with small values of δ, see above.

Fig. S6 shows the spreading of the perturbations extracted from the distance d with a threshold $\delta = 1.8$. The four panels corresponds to four ensembles of perturbations where only one type of bead is poked (all beads are related by a lattice translation). These measurements show that the structure is composed of 3×4 molecules, all of them being revealed by the purple, blue, and green perturbations. However, the yellow perturbations only define 3×3 polygonal regions. The chiral molecules are defined by the ensemble of beads and springs lying inside the superposition of all the polygonal regions. Correctly pairing the perturbations amounts to superimposing the colored polygons and looking for the largest overlaps. In this case, the yellow polygons then contribute to the last three columns of molecules only. The left edge of the sample is made of mechanical molecules that are distinct from the bulk molecules as clearly seen in Fig. 1d.

Fig. 1d reflects the final mechanical molecules. We then proceed as usual: we compute the average centers and the average chiral polarization field, see Fig. 1e.

For the numerical study of the checkerboard lattice in Fig. 4 we use $\delta = 1.8$ over 20 time steps to avoid finite-size effects.

3. Extended Figures

In this section we provide the complementary plots and measurements obtained from experiments and simulations.

A. Robustness of the local dynamics as proxies of the Wannier functions: 1D linear simulations. Fig. S7 shows the chiral centers and chiral polarization for distinct perturbations with increasing amount of noise for a linear simulation of the one-dimensional chain of rotors and springs.

Fig. S8 shows the chiral centers and chiral polarizations issued from the Wannier functions in different systems interpolating from the atomic limit (rotors and spring aligned) to the conducing system (rotors at $\pm\pi/2$ angle). Except from the conductor, which has a null polarization, all the other cases exhibit a polarization of $\Pi_x = 0.5a$, with a being the unit-cell length.

Fig. S9 shows illustrates the same information as fig. S8, this time using identical localized Gaussian functions. The conclusions are the same.

B. Experiments in the mechanical chain. Fig. S4 illustrates the raw displacements and elongations of all the nodes for the one-dimensional mechanical chain of rotors.

Fig. S10 shows the norm, the center, the chiral charge, and the chiral polarization for each perturbation in the mechanical chain. The data shown are those for which norm is larger than $\epsilon_{exp} = 0.3$.

Fig. S11 illustrates the dependence of the time-averaged values on the period Δt. In the main text we chose $\Delta t = 80$ms.

C. FEM simulations of the mechanical chain. Following the same structure, we show the raw data (Fig. S12), the moments (Fig. S13) filtered by $\epsilon_{textFEM} = 0.01$, and the dependence of the time averages on the period Δt (Fig. S14). For the results shown in the main text, we use $\Delta t = 2$ms. As opposed to the experiments, the FEM simulations do not take into account any damping.

D. Experiments in the two-dimensional metamaterial. Fig. S15 shows the norm, the center, the chiral charge, and the chiral polarization for each perturbation in the two-dimensional metamaterial. We only show the data for which the norm is larger than $\epsilon_{\mathrm{FEM}} = 0.6$.

Fig. S16 illustrates the dependence of the time-averaged values on the period Δt. In the main text we chose $\Delta t = 30$ms.

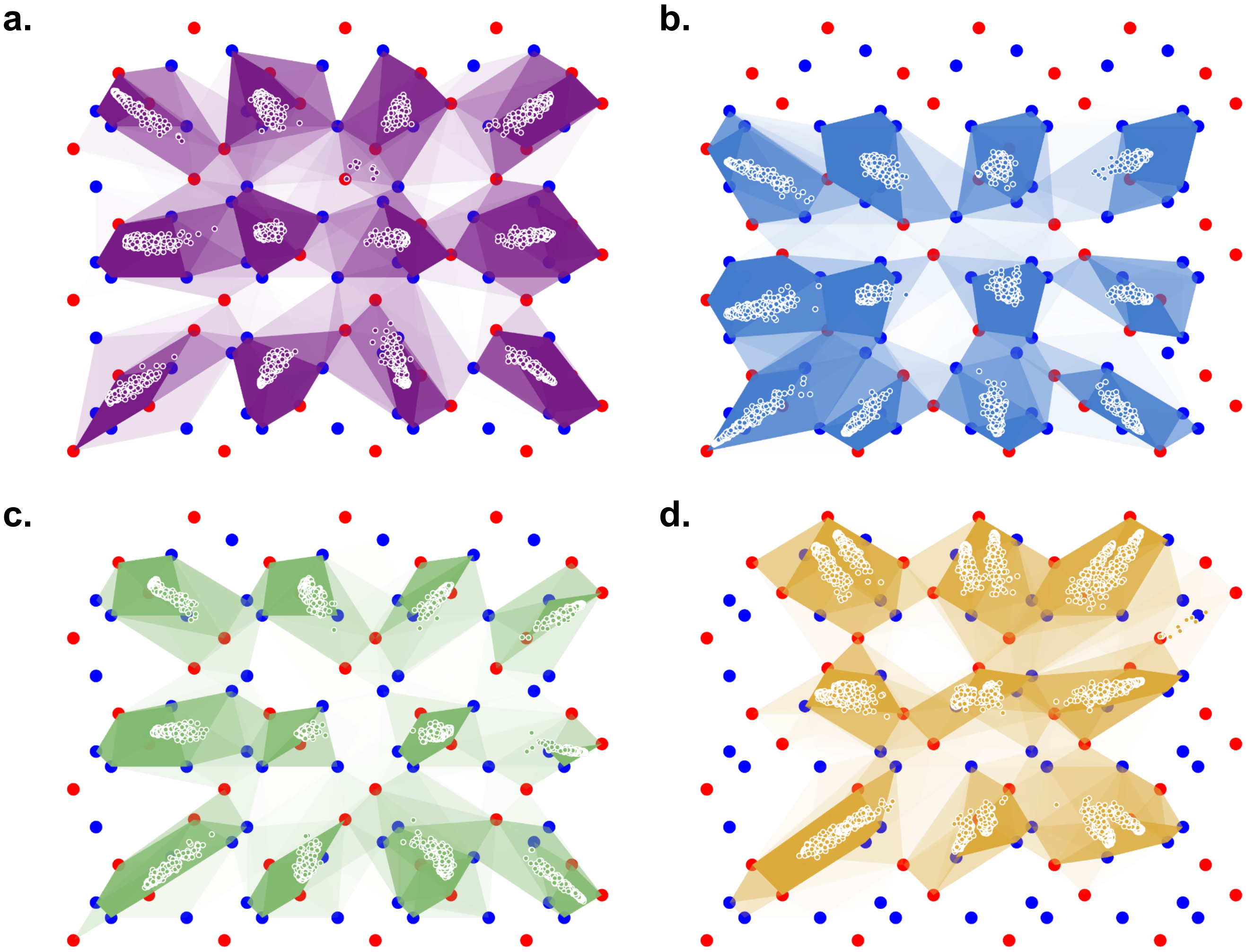

Fig. S6. **Mechanical molecules in the experimental poking of 2D mechanical system.** For each perturbation $\Psi(t)$ we draw the polygons spanning all the sites (red for beads, blue for springs) for which $d < \delta = 0.8$. Each polygon, of light opacity, is superposed as time evolves. Opaque regions thus distinguish the strongly connected sites. Each panel ($\mathbf{a}$ to $\mathbf{d}$) contains all the polygons associated to the perturbation of the same bead in different unit cells. We also highlight the centers $\boldsymbol{r}(t)$ of the perturbations as points of the same colors as the polygons.

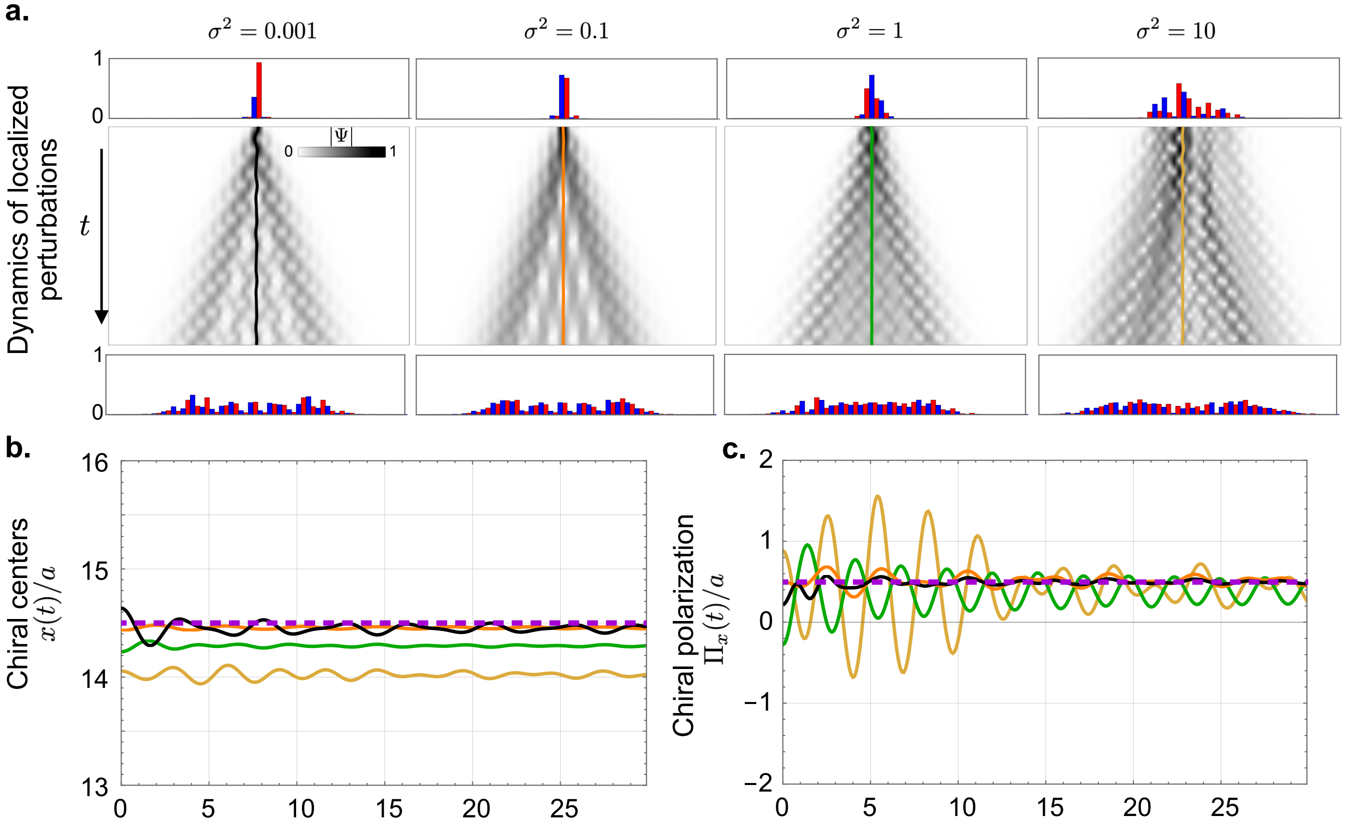

Fig. S7. Robustness of the local dynamics as proxies of the Wannier functions. a. Evolution of four distinct localized perturbations in a one-dimensional mechanical chain of 31 rotors. The initial conditions are centered in the middle of the finite system. All the perturbations are drawn from a Wannier function W with noise proportional to a Gaussian function centered around the midpoint of the chain, $\exp\left(-(x - x_{\text{center}})^2/\sigma^2\right)$. From left to right: Linear evolution of the localized initial conditions for increasing values of the spreading of the perturbation σ^2. In all three linear cases the initial state spreads with at a rate fixed by the stiffness and the mass of the beads $\sqrt{k/m} = 1$. At each time step we compute the chiral center $\mathbf{b}$ and the chiral polarization $\mathbf{c}$, both of them normalized by the unit-cell length a. The purple dashed line corresponds to the theoretical case of a Wannier function.

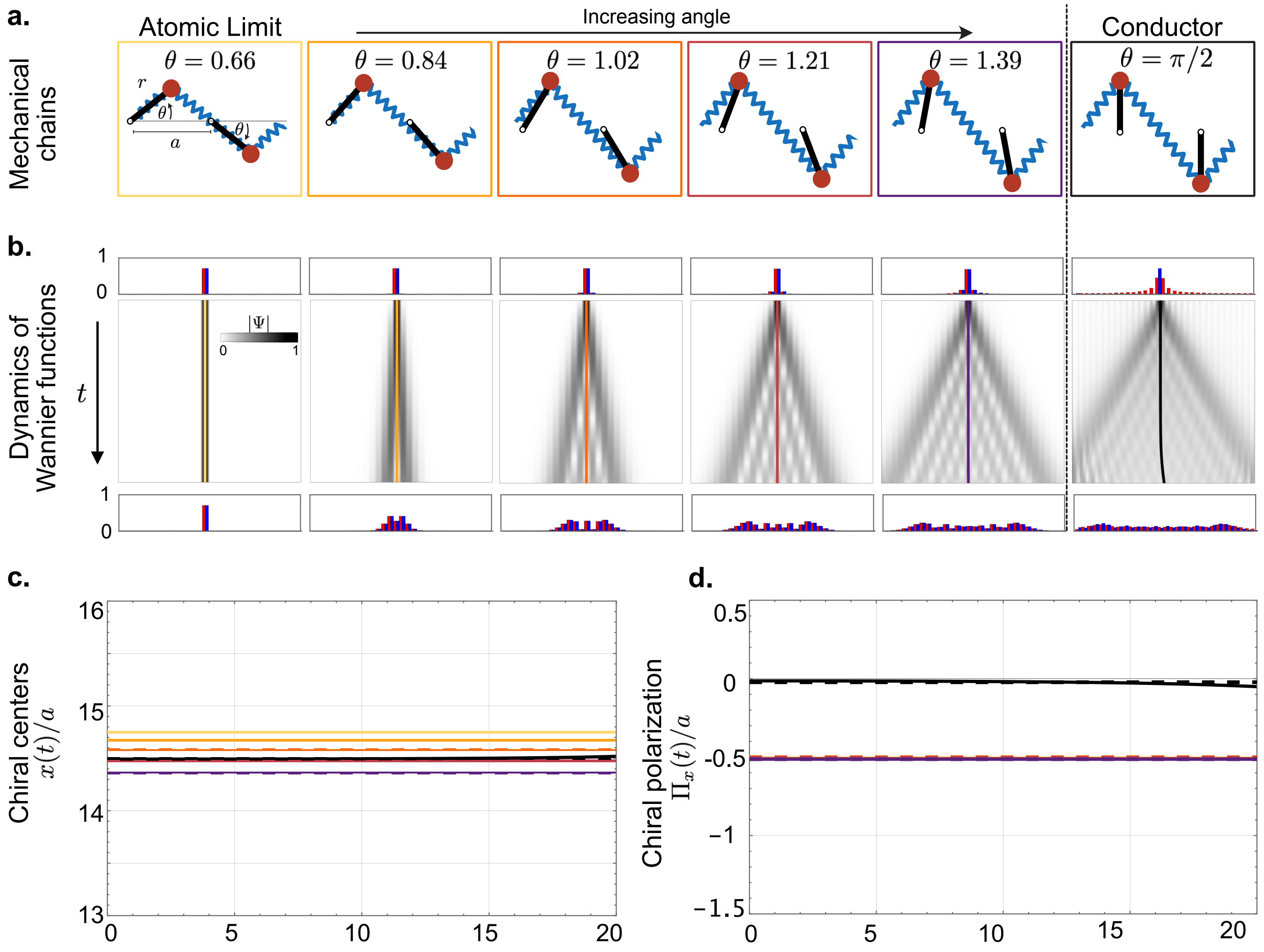

Fig. S8. From the atomic limit to conductor in the 1D chain: Wannier functions a. Six different realizations of the Kane-Lubensky differing by the inclination angle θ formed by the rotor with the horizontal axis. In all cases $r/a = 0.63$. In the atomic limit rotors and springs are colinear and therefore a given angular displacement only compresses the right spring. Increasing the angle diminishes the asymmetry of the response, up to the point in which the system becomes a conductor, $\theta = \pi/2$. **b.** (top) Wannier functions located in the middle of the sample. As the angle increase, the initial functions have a wider support until becoming completely delocalized. (middle) Evolution of the Wannier functions, with its center highlighted by a colored solid line. The spreading increases with the angle. In the case of the atomic limit, the evolution remains localized to the unit cell compatible to the atomic limit (bottom). **c.** Centers of the Wannier function in time (solid) with its time average (dashed) for each case in different colors. **d.** Chiral polarization of the Wannier functions. For all first five cases, the polarization is the same and the curves overlap. For the case of a conductor, the polarization is zero.

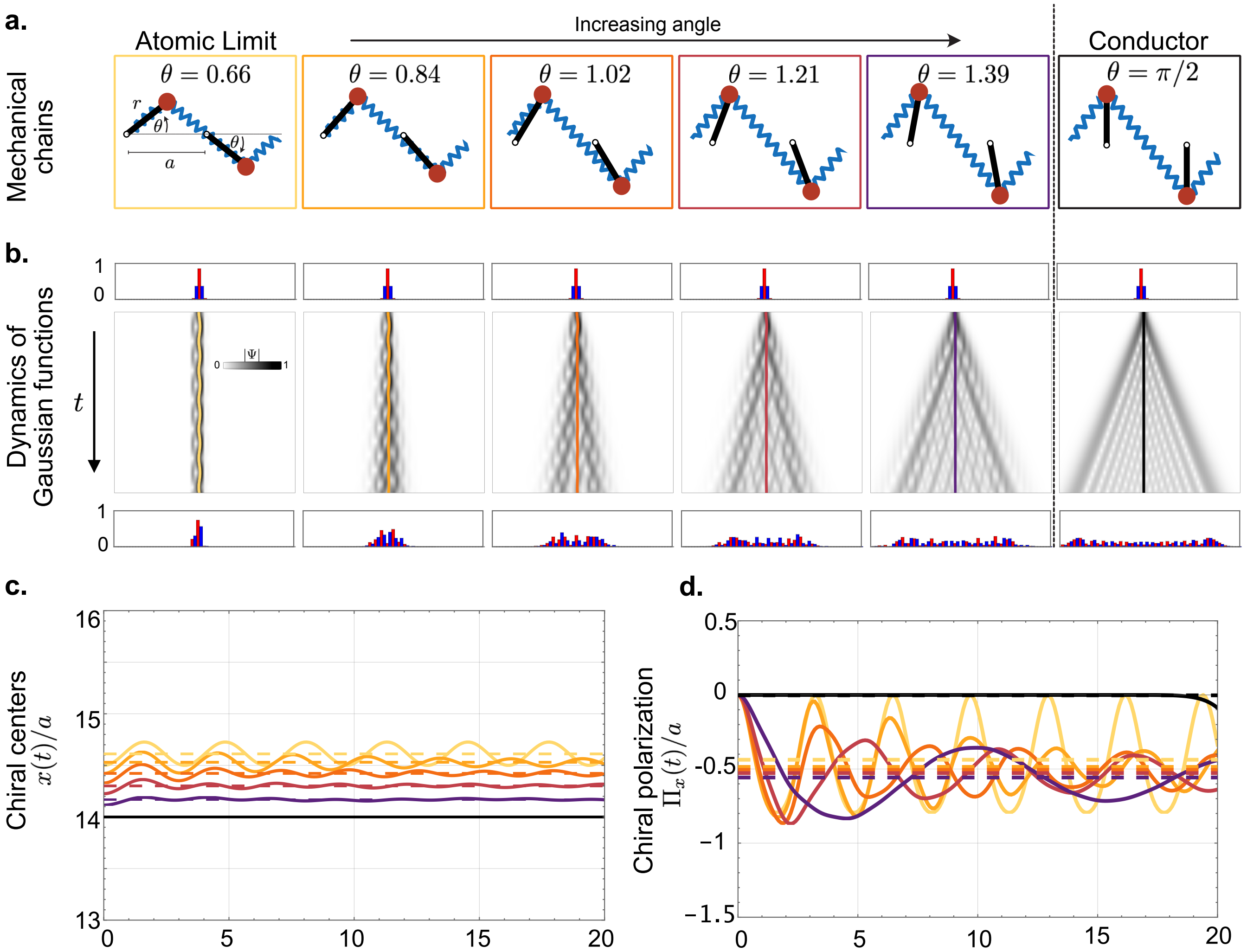

Fig. S9. **From the atomic limit to conductor in the 1D chain: Gaussian functions** Same as fig. S8, this time using the same Gaussian function as an initial state. An increasing angle (**a**) leads to a more spread evolution (**b**). Both the centers (**c**) and the polarizations (**d**) oscillate in time. However, their time average coincide for the first five cases, giving a polarization $\Pi_x = 0.5a$ with a the unit-cell length. This drastically changes when dealing with a conductor (black line), for which the polarization is $\Pi_x = 0.$.

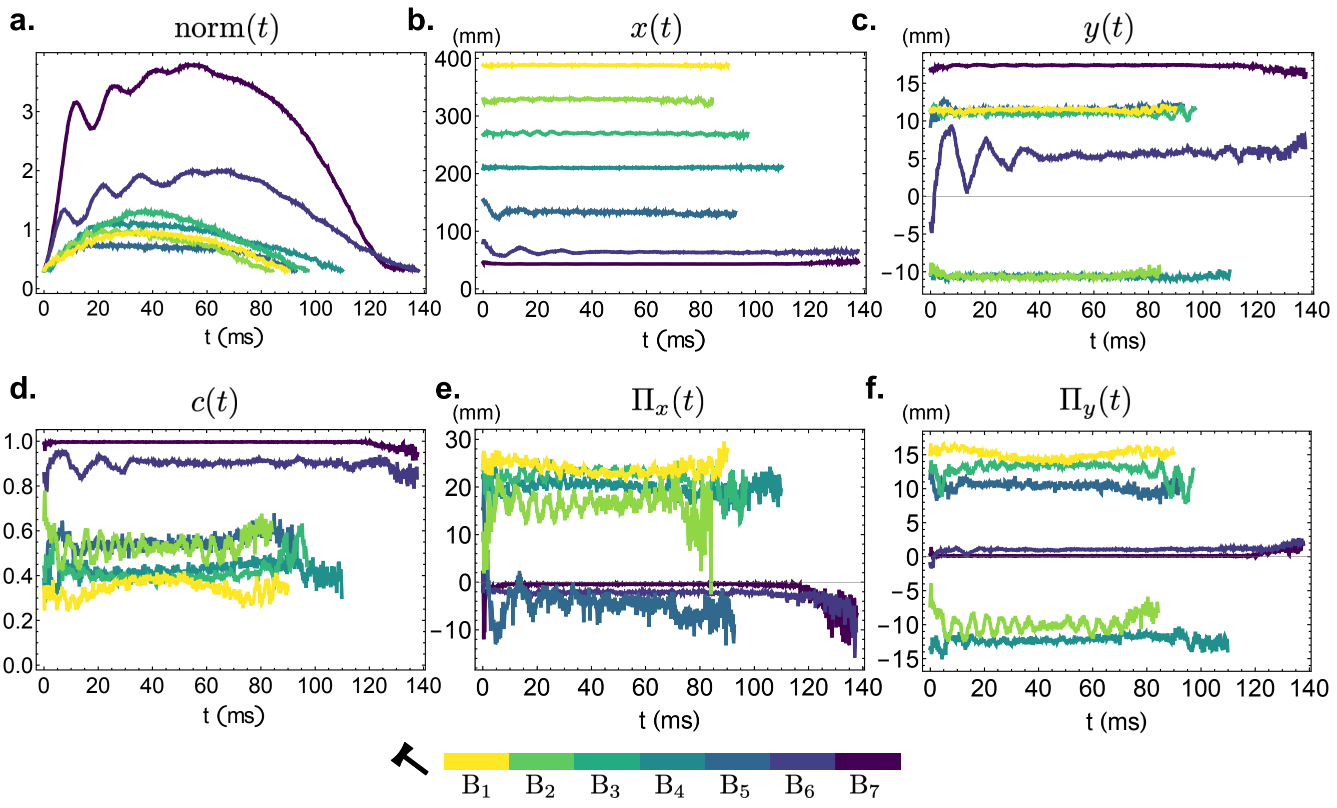

Fig. S10. **Moments from local perturbations in the mechanical chain (Experiments).** Norm (**a**), positions (**b** and **c**), chiral charge (**d**) and chiral polarization components (**e** and **f**), for all the different perturbations in the mechanical chain (color).

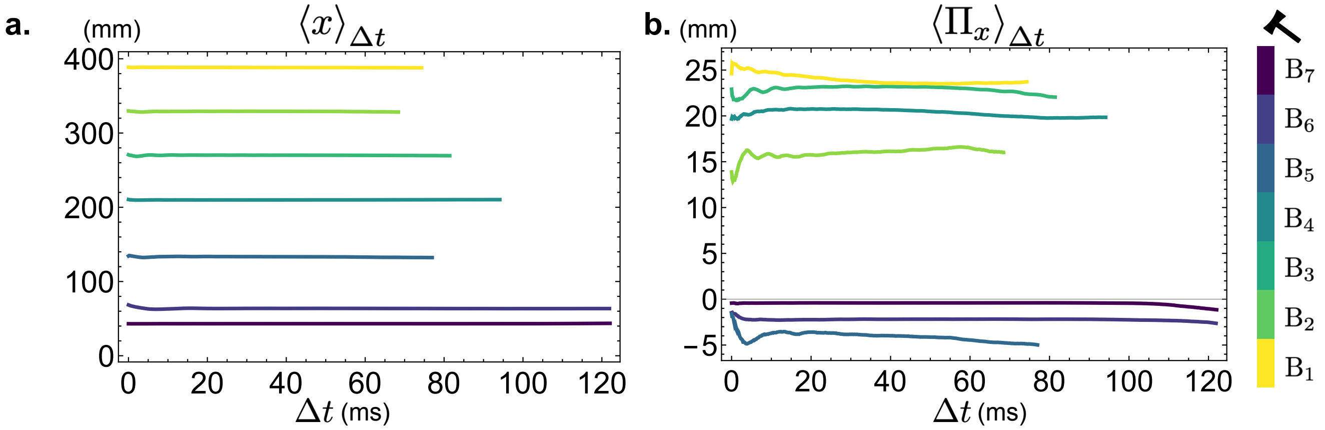

Fig. S11. **Time-averaged moments in the mechanical chain (Experiments). a.** Due to the constant-like signal of the horizontal position (Fig. S10b), the time-averaged horizontal position, $\langle \boldsymbol{x} \rangle_{\Delta t}$, is independent of the period Δt. **b.** The average chiral polarization, $\langle \Pi_x \rangle_{\Delta t}$ is weakly dependent on Δt yet the differences remain much smaller than the unit cell length $a = 60$mm.

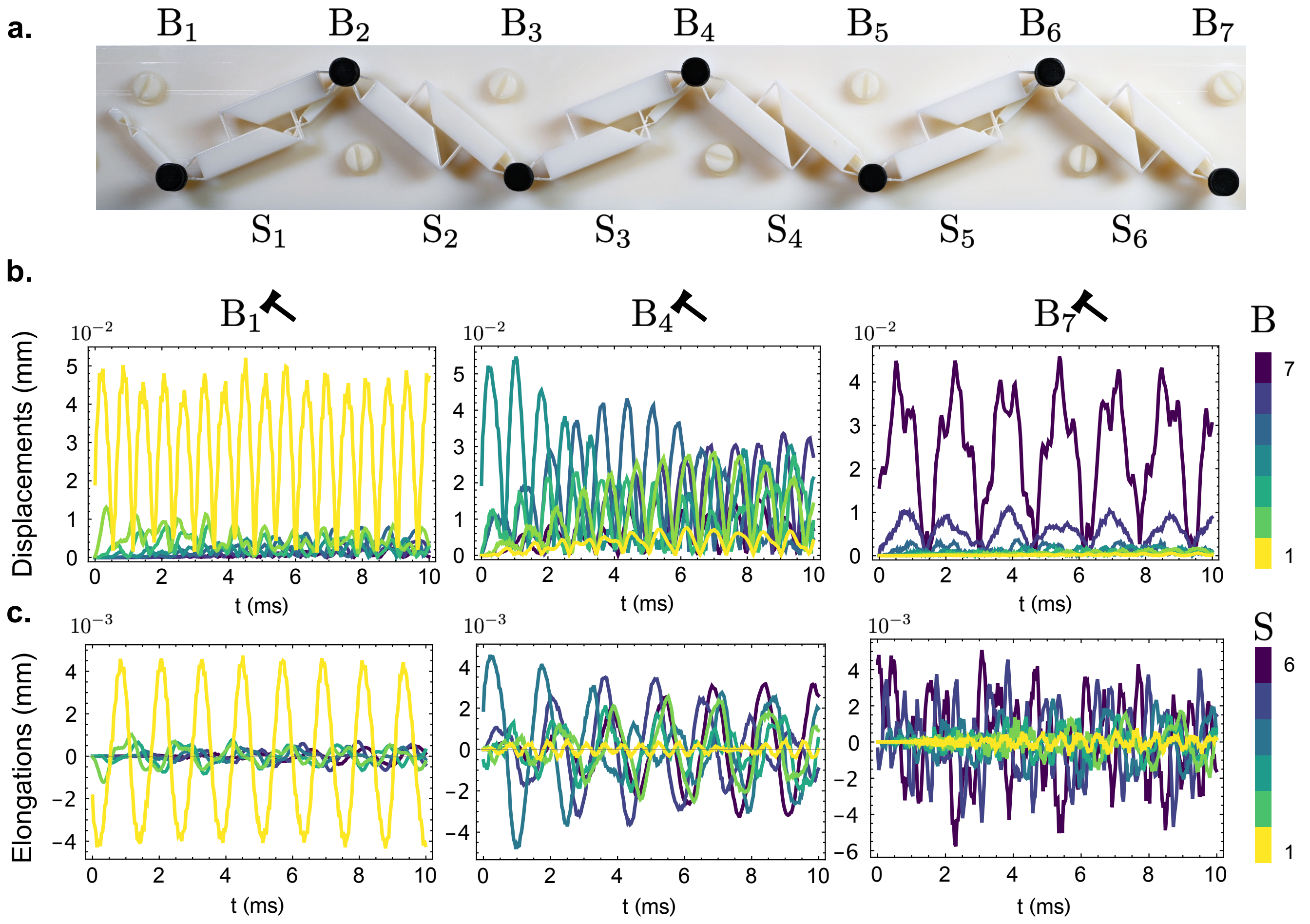

Fig. S12. Raw displacements and elongations from local perturbations in the mechanical chain (FE simulations). (top) Displacement of each node (color) when hammering the first (left), fourth (middle) and seventh (right) node. (bottom) Corresponding elongations for all the springs (color).

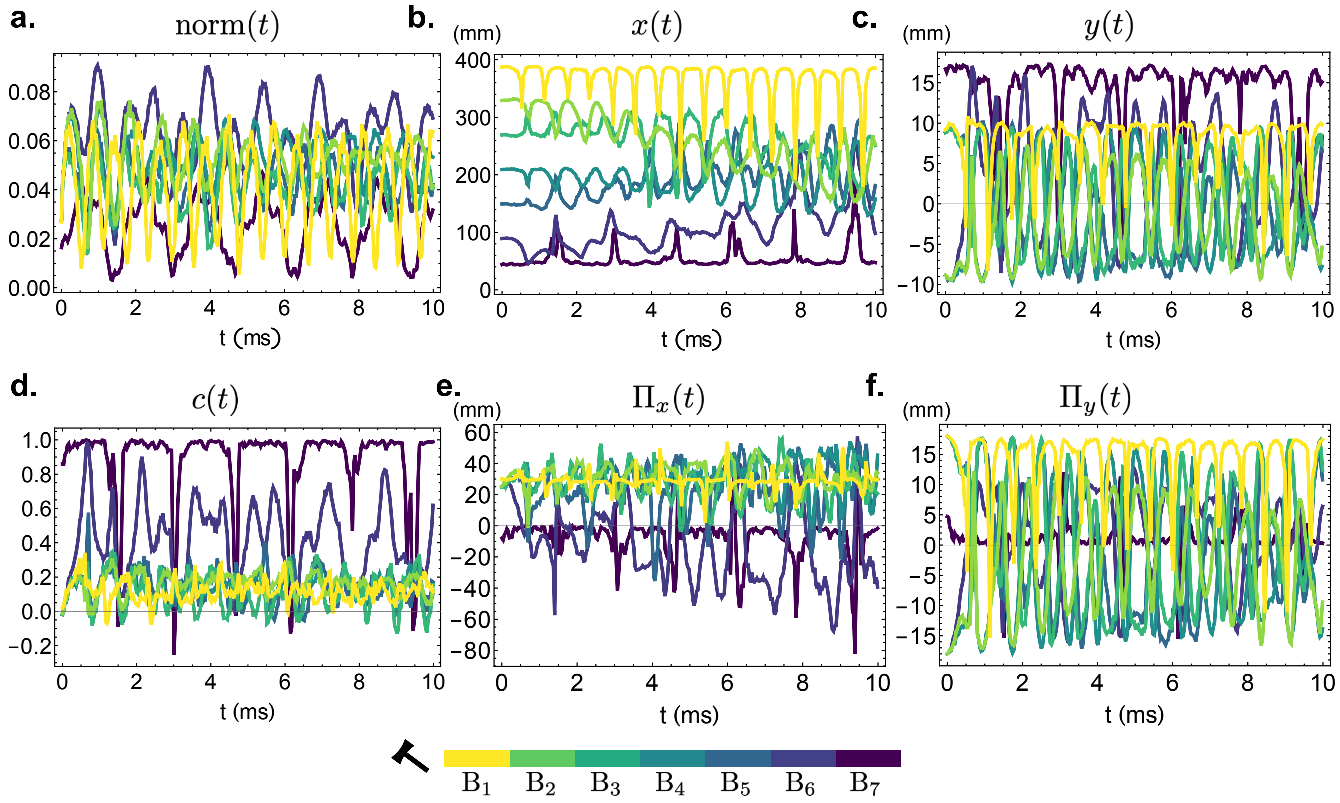

Fig. S13. **Moments from local perturbations in the mechanical chain (FE simulations).** Norm (**a**), positions (**b** and **c**), chiral charge (**d**) and chiral polarization components (**e** and **f**), for all the different perturbations in the mechanical chain (color). After 100 ms from the impulsive perturbation, the response is completely dampen and the signal is noise-dominated.

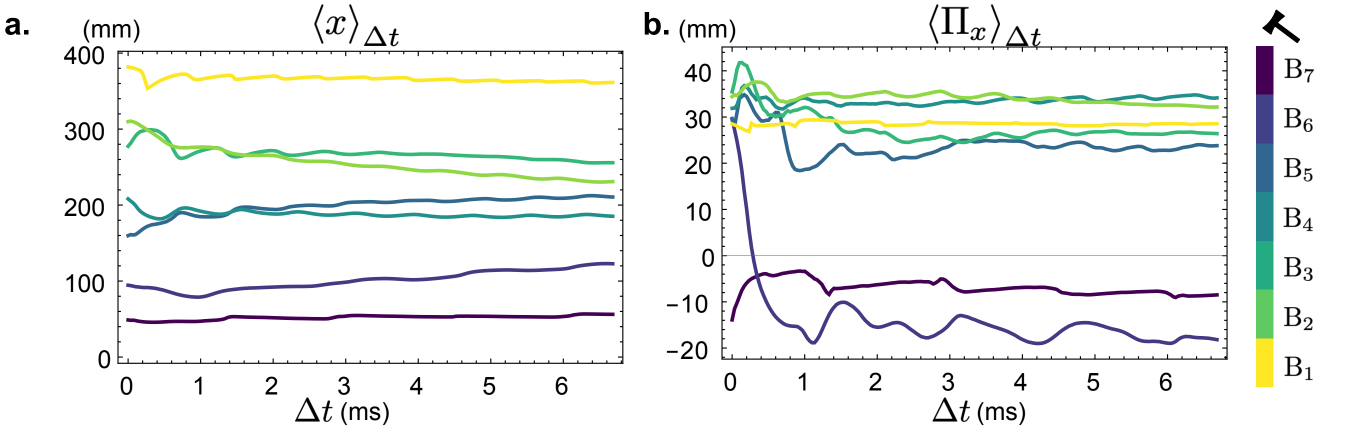

Fig. S14. Time-averaged moments in the mechanical chain (FEM simulations). Time-averaged horizontal position (**a**) and polarization (**b**) for each perturbation. In both cases, due to the absence of friction, the averages are more dependent on the period Δt than they are in the experiments.

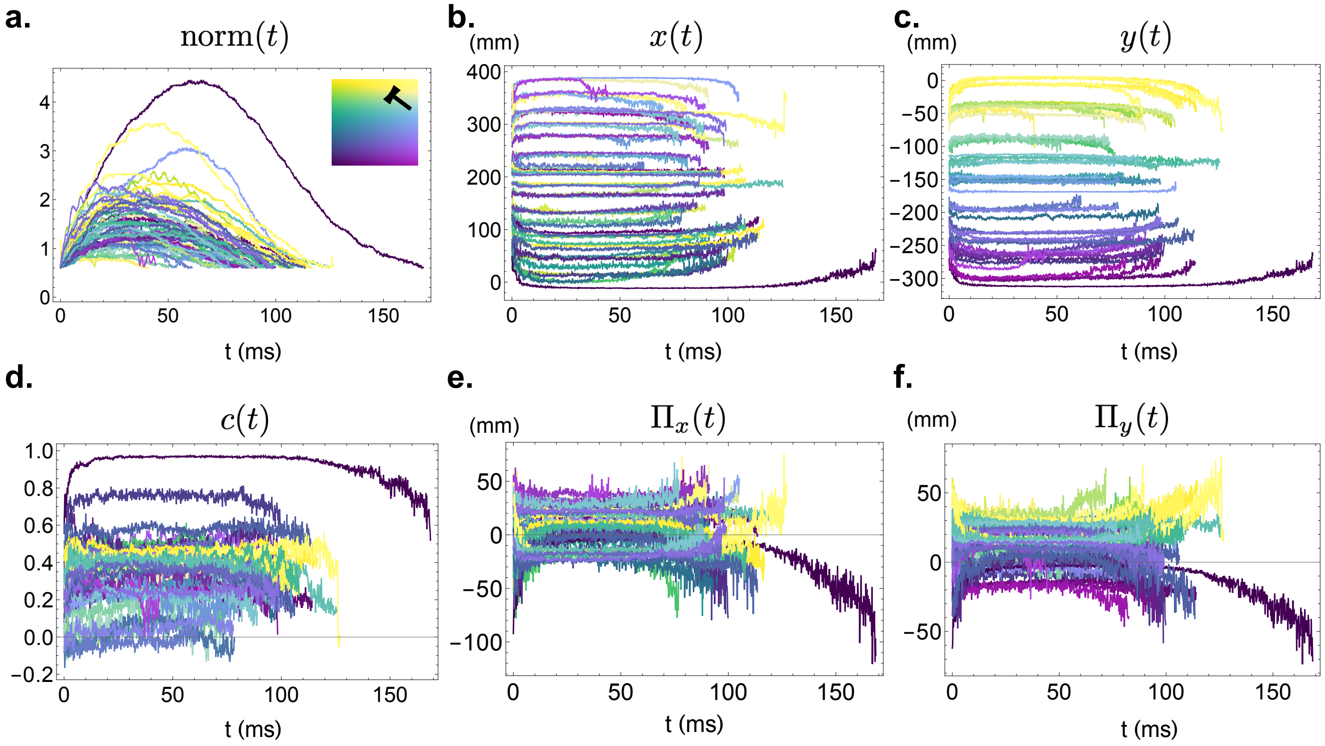

Fig. S15. Moments from local perturbations in the mechanical two-dimensional metamaterial (Experiments). Norm (**a**), positions (**b** and **c**), chiral charge (**d**) and chiral polarization components (**e** and **f**), for all the different perturbations in the mechanical metamaterial (colormap in the inset of **a**). Only the data for which norm$(t) > \epsilon_{exp}$ is considered.

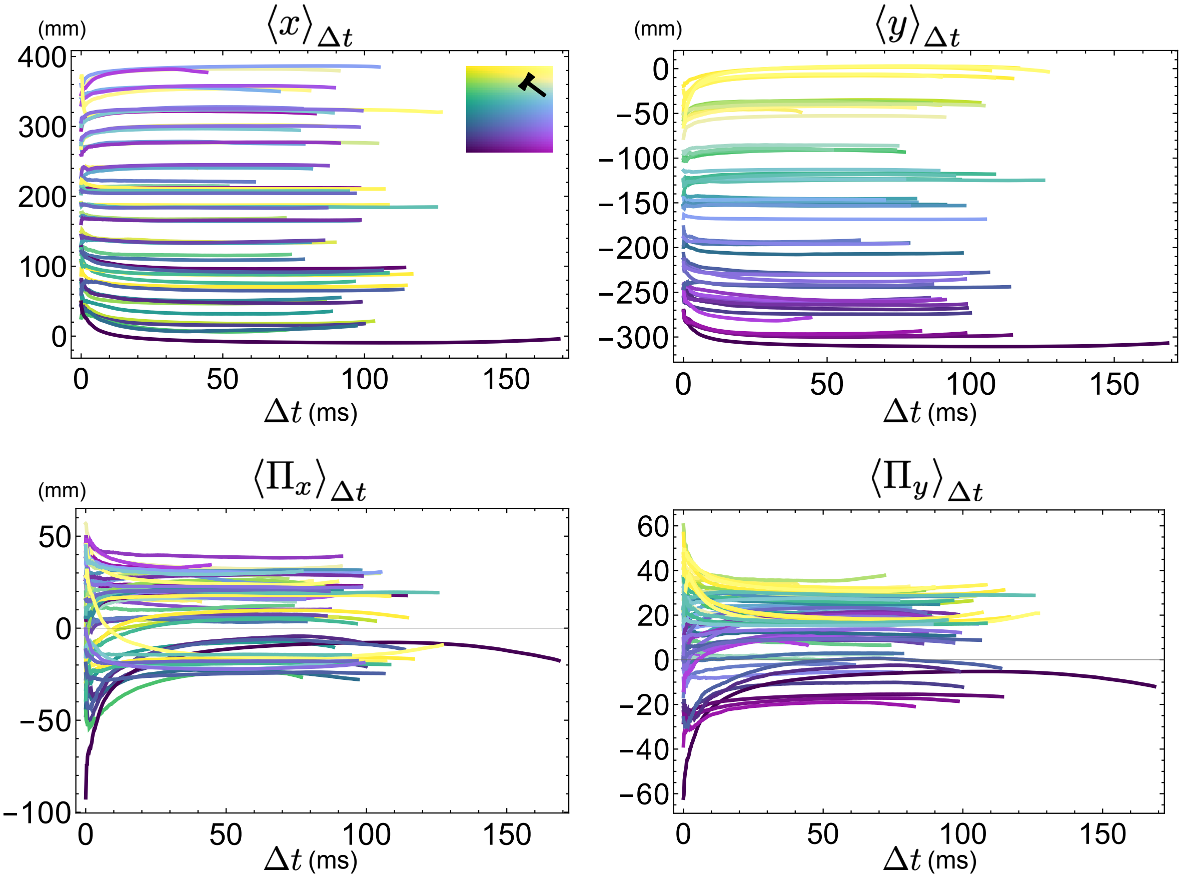

Fig. S16. **Time-averaged moments in the mechanical two-dimensional metamaterial (Experiments).** Vertical and horizontal components of the average center and chiral polarization as a function of the period Δ_t. Each color represents a different perturbation according to the colormap shown in the inset of the first panel.

E. FEM simulations of the two-dimensional metamaterial. Following the same structure, we show the moments (Fig. S17) filtered by $\epsilon_{textFEM} = 0.01$, and the dependence of the time averages on the period Δt (Fig. S14). For the results shown in the main text, we use $\Delta t = 0.5$ms.

Fig. S19a to d. shows the extent of the perturbations for each type of node in a unit cell. Fig. S19e and f reveal the final mechanical molecules and chiral polarization field of the simulated system.

4. Sample design

A. Basic units. Inspired by the mechanical chain of ref. (2), the designs of the mechanical metamaterials presented in our paper are based on two basic units, respectively called rotors and springs (see Fig. S20 (a) and (b)). Unlike the ideal model presented by Kane and Lukensky, the rotors and springs we present are not made from pure mechanisms, but from compliant mechanisms. Therefore their deformation does not cost zero elastic energy but a finite level of elastic energy (3, 4). We optimize the geometrical parameters of our compliant mechanisms such that the first eigenmode of the rotor is a rotation with respect to the pivot s, and the first eigenmode of the spring is an axial compression/elongation, see Fig. S20 (c). We further optimize the design by requiring that higher eigenmodes have much higher eigenvalues. Therefore, at low frequencies the target compliant mechanisms will dominate. These constraints typically require the use thin-walled hinges. However, there is a trade-off and to guarantee the printing quality, the minimum thickness of thin-wall structures is set to 0.48 mm.

We conducted an analysis of the eigenmodes of mechanical metamaterials comprising N rotors. We found that for the first N eigenmodes of the mechanical metamaterial, all rotors deform as rotations and all springs deform as elongations or shortening. All deformations closely follow those prescribed by the compliant mechanism and no spurious mode pollutes the spectrum. In this article, we adopt the parameters shown in Fig. S20 (b).

B. Mechanical chain. Here we detail the design of the mechanical chain used in the Main Text. We begin with a solid optical panel on which we fix the 3D printed structure, see Fig. S21(a). The dimensions of the panel are $L_1 = 420$ mm, $H_1 = 72$ mm and $t_1 = 4.8$ mm. The black strip on the panel helps to calibrate the camera angle. In order to improve the image analysis and data acquisition, we 3D print black disks on the rotors' tip, see Fig. S21(a). The pivots' positions are horizontally aligned and equally spaced by $a = 60$ mm. The angle between the rotors at equilibrium and the horizontal line is $\theta_0 = \pm\pi/4$. By design, rotors and springs will overlap with each other when assembled together. Therefore, we put rotors and springs in different planes. The 1D chain has four layers in total, see Fig. S21 (a). The bottom layer is the solid panel, the second layer hosts the rotors, the third one hosts the springs and the final one hosts the black disks. The pivots of the rotors are fixed on the solid panel (shown as junction 1) and springs connect the end of adjacent rotors (shown as junction 2).

The gap between the rotors and the solid panel along the z direction is 1.2 mm, the gap between the rotors and the springs is 0.12 mm and the gap between springs and beads is 0.12 mm. The gap between the layers are small to reduce influence of the out-of-plane deformation.

To implement a domain wall in the chain, we just need to change the tilt angle of some rotors. For a floppy mode domain wall, we connect a left a chain with a tilt-rotor angle $\theta_{\mathrm{left}} = \pm\pi/4$ to a right chain with tilt angle $\theta_{\mathrm{right}} = \pm 3\pi/4$, through a vertical middle rotor ($\theta_{\mathrm{middle}} = \pi/2$), see Fig. S21(b). For a self-stress state domain wall, we interchange left and right chains while keeping the same middle rotor, see Fig. S21(c).

C. Two-dimensional metamaterial. The two-dimensional metamaterial assembles the 3D printed rotors and springs of Fig. S20, and in addition, single beads. This corresponds to connecting two springs via a black bead with no rotor, see Fig. S22. The unit cell of the metamaterial hosts 3 rotors, 4 springs, and 1 free bead, see Fig. S22. The whole metamaterial is made out of 3.5 units along the horizontal direction and 3 units along the vertical direction. The dimensions of the solid panel are $L_3 = 480$ mm and $H_3 = 384$ mm. Due to the higher connectivity of the network, the springs are located in two different layers, see Fig. S22.

5. Sample fabrication

All specimens are fabricated by additive manufacturing using a PolyJet 3D printer (Stratasys Object500 Connex3), whose build area is 490 mm × 390 mm × 200 mm. The accuracy of the printer is 200 microns. The whole specimen is made of the same photopolymer, Stratasys Vero (Young's modulus $E \approx 2500$ MPa). To improve the image analysis and data acquisition, we use a black material (Veroblack) for the beads and the calibration strip, and a white material (Verowhite) for the rest.

6. Experimental setup and data acquisition

A. Data acquisition. To measure the chiral polarization, we need to perturb each bead, or rotor-tip, and measure the displacement of the black beads. To track the positions of beads, we record the experiments using a high-speed camera (Phantom VEO 640). We record all the tests with 6300 fps, and a resolution of 1792 px × 480 px for the mechanical chains and 1024 px × 768 px for the two-dimensional metamaterial. We use the particle detection and tracking function in ImageJ to analyze the images and acquire the positions of the beads. We first threshold images (replace each pixel with black or white pixels) and then track the center of each pattern of black pixels. Each bead has around 18 to 35 pixels along the diameter. The accuracy of the measurement is around 0.006 mm (0.015 pixels).

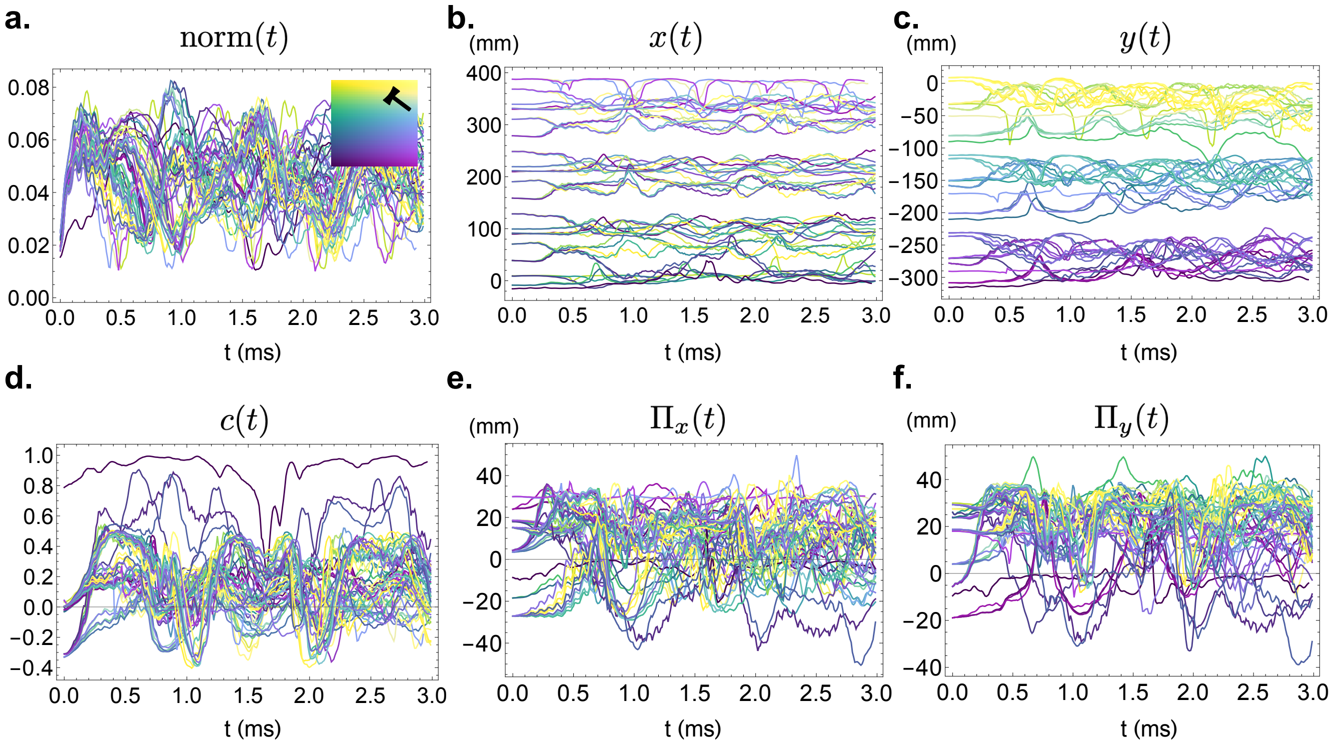

Fig. S17. **Moments from local perturbations in the mechanical two-dimensional metamaterial (FEM simulations).** Norm (**a**), positions (**b** and **c**), chiral charge (**d**) and chiral polarization components (**e** and **f**), for all the different perturbations in the mechanical metamaterial (colormap in the inset of **a**). Only the data for which norm$(t) > \epsilon_{FEM}$ is considered.

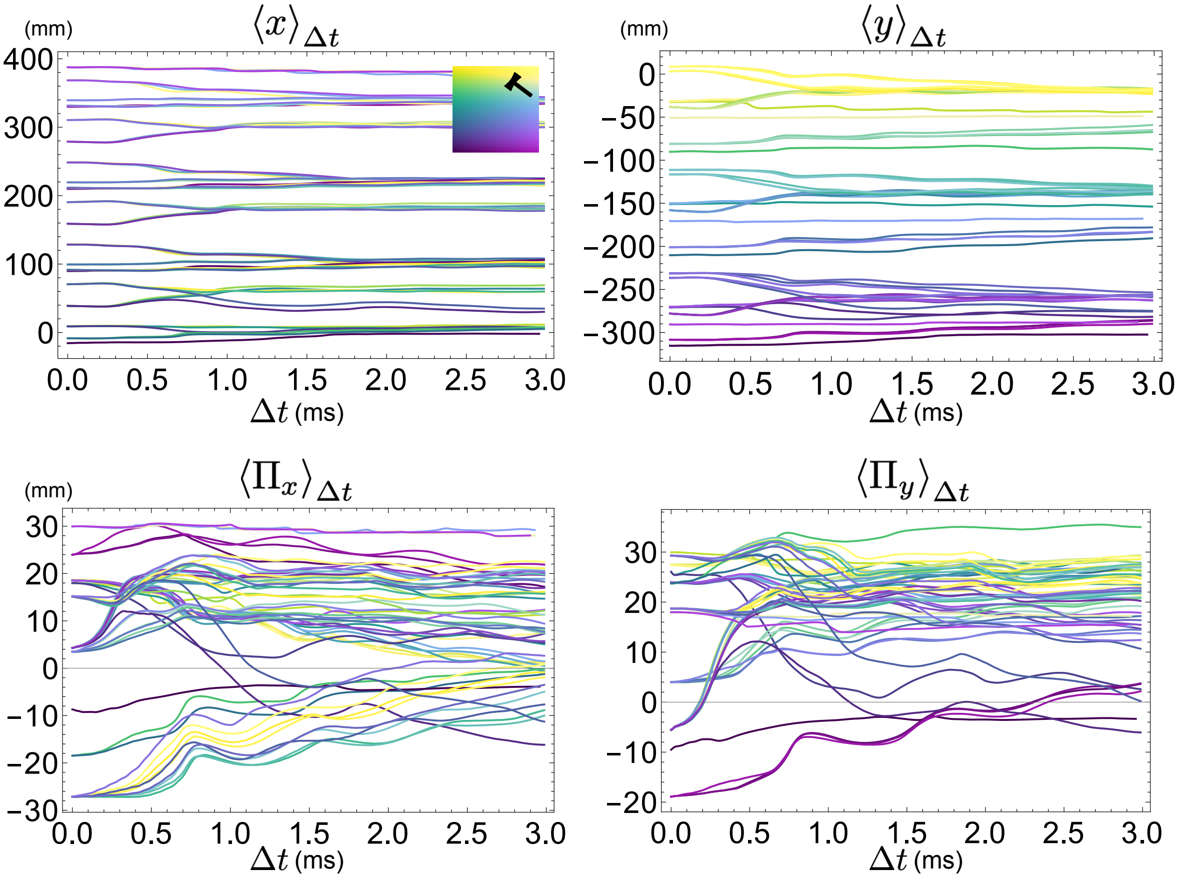

Fig. S18. **Time-averaged moments in the mechanical two-dimensional metamaterial (FEM simulations).** Vertical and horizontal components of the average center and chiral polarization as a function of the period Δ_t. Each color represents a different perturbation according to the colormap shown in the inset of the first panel.

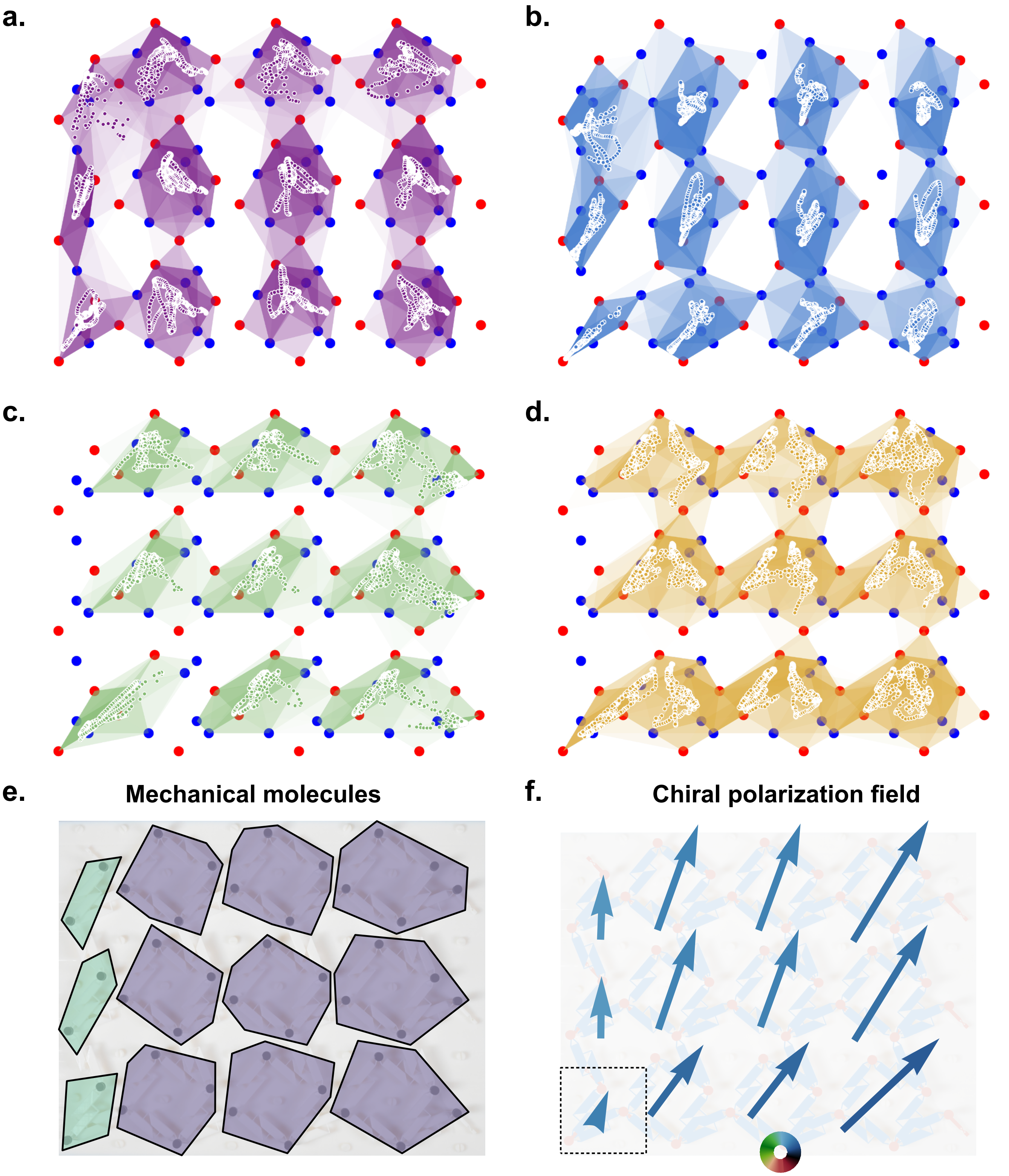

Fig. S19. Mechanical molecules in the simulated poking of 2D mechanical system. For each perturbation $\Psi(t)$ we draw the polygons spanning all the sites (red for beads, blue for springs) for which $d < \delta = 1, 2$. Each polygon, of light opacity, is superposed as time evolves. Opaque regions thus distinguish the strongly connected sites. Each panel (**a** to **d**) contains all the polygons associated to the perturbation of the same bead in different unit cells. We also highlight the centers $\boldsymbol{r}(t)$ of the perturbations as points of the same colors as the polygons. **e.** As in the experimental poking (see Fig. 1d of the main text) there are two types of mechanical molecules: large ones in the bulk (violet) and smaller ones in the left edge (green). **f.** The chiral polarization exhibits the same discontinuity at the bottom left corner (dashed square).

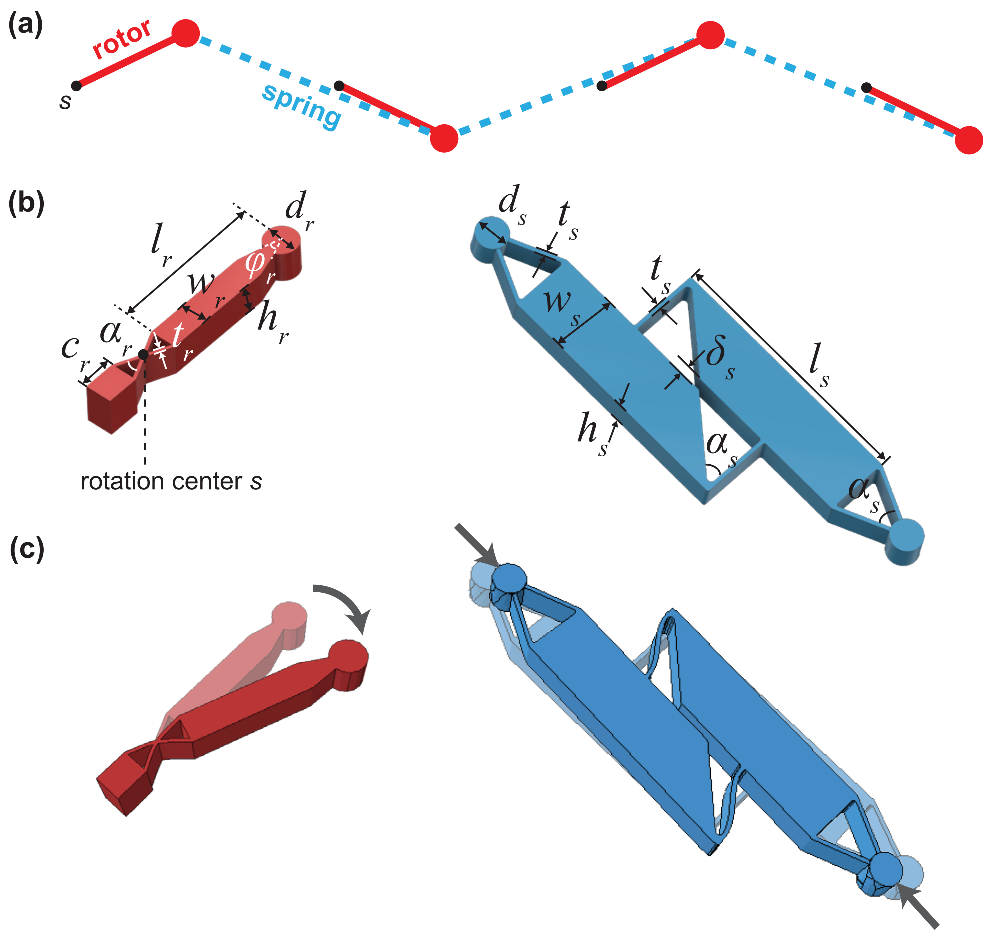

Fig. S20. Basic units of the mechanical metamaterials. (a) Ideal model of a 1D mechanical SSH chain. **(b)** Design of two basic units: rotor (red) and spring (blue), where $l_r = 21.9$ mm, $w_r = 3.9$ mm, $h_r = 4.8$ mm, $t_r = 0.48$ mm, $d_r = 4.8$ mm, $\alpha_r = 50^\circ$, $\varphi_r = 31.5^\circ$, $c_r = 4.8$ mm, $l_s = 37$ mm, $w_s = 9.6$ mm, $h_s = 4.8$ mm, $t_s = 0.6$ mm, $d_s = 4.8$ mm, $\alpha_s = 50^\circ$ and $\delta_s = 1.2$ mm. **(c)** Illustration of the lowest energy eigenmode of both units. For a rotor (left), it corresponds to a rotation with respect to the pivot s, whereas for a spring it corresponds, primarily, to an axial compression (right).

B. Perturbation experiments. For the perturbations, we use a transparent plastic wire to pull the beads. We use the transparent wire for the following two reasons: i) the transparent wire is almost invisible on the photo and therefore improves the image analysis. ii) The direction of the perturbation is easier to control. To simplify the pulling, we design a groove on beads, see Fig. S20(a). The displacement applied is around 1-2 mm in a direction roughly perpendicular to the rotor. Since the displacements are very small, the vibration of the whole system during the experiment can significantly affect the final data. To improve the steadiness, we screw the system to an optical panel attached to a steady table.

C. Vibration experiments. To observe the floppy corner mode of the two-dimensional metamaterial, we conducted vibration experiments by mounting the specimen onto an optical panel attached to a vibration machine (Tira Vibration Test System TV 5220-120). The vibration machine generated vertical vibrations that follow a sine wave function. We adjust the frequency using an Aim-TTi TG5011 function generator. The first mode appears at a vibration frequency of 88Hz.

7. Numerical simulations

We conducted finite element simulations using the commercial package ABAQUS. To capture the dynamic evolution, we employed the explicit solver, while the standard solver was used to acquire the eigenmodes. We discretize the model with tetrahedrons (20-node quadratic brick with reduced integration, element type: C3D20R). Each pair of rotor and spring is divided in 2000 to 3000 tetrahedrons. The simulated systems are composed of a linear elastic material with Young's modulus $E = 2500$MPa, Poisson's ratio $\nu = 0.33$, and density $\rho = 1.19$ g/cm^3 (from Stratasys Vero product data sheet). The rectangle panel is fixed. Out-of-plane deformations are also constrained in all simulations. The perturbation is given by displacement loads perpendicular to the rotor $|dx| = 0.01$ mm and $|dy| = 0.01$ mm within 0.0001s (smooth step). Then, the system is released, and we track the displacements of all nodes for a duration of 0.004s to 0.01s.

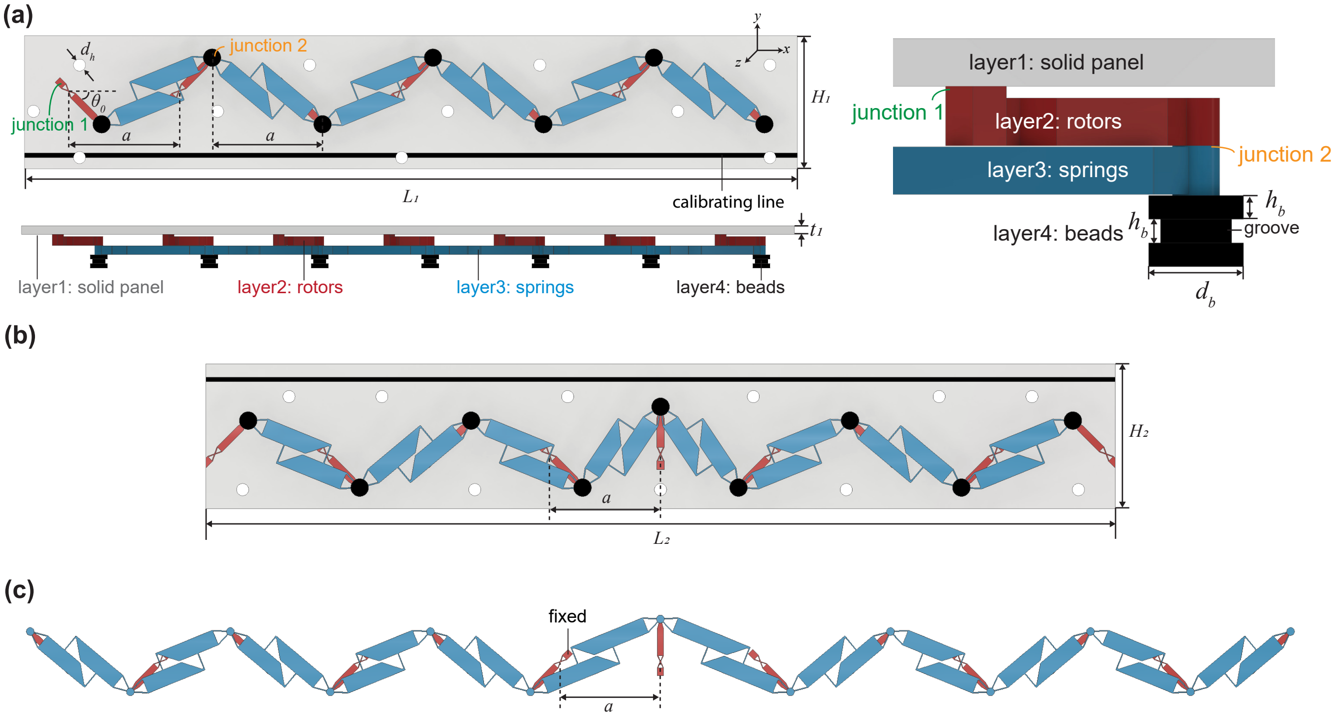

Fig. S21. **Design of the mechanical chains.** **(a)** Front view (top left) and top view (bottom left and right) of a homogeneous mechanical chain. The size of the panel is $L_1 = 420$ mm, $H_1 = 72$ mm, and $t_1 = 4.8$ mm. The diameter of screw holes is $d_h = 6.5$ mm. The rotation centers of the rotors are fixed on the solid panel (junction 1). The rotor layer and spring layer are connected at junction 2. The dimensions of beads are $d_b = 9.6$ mm and $h_b = 2.4$ mm. **(b)** Design of a heterogenous mechanical chain with a floppy mode in the middle. The panel size is $L_2 = 489.6$ mm and $H_2 = 78$ mm. **(c)** Design of a heterogeneous mechanical chain with a self-stress state domain wall. This model is designed for Finite Element Method (FEM) simulations. Here we prescind from both the solid panel and the bead layer.

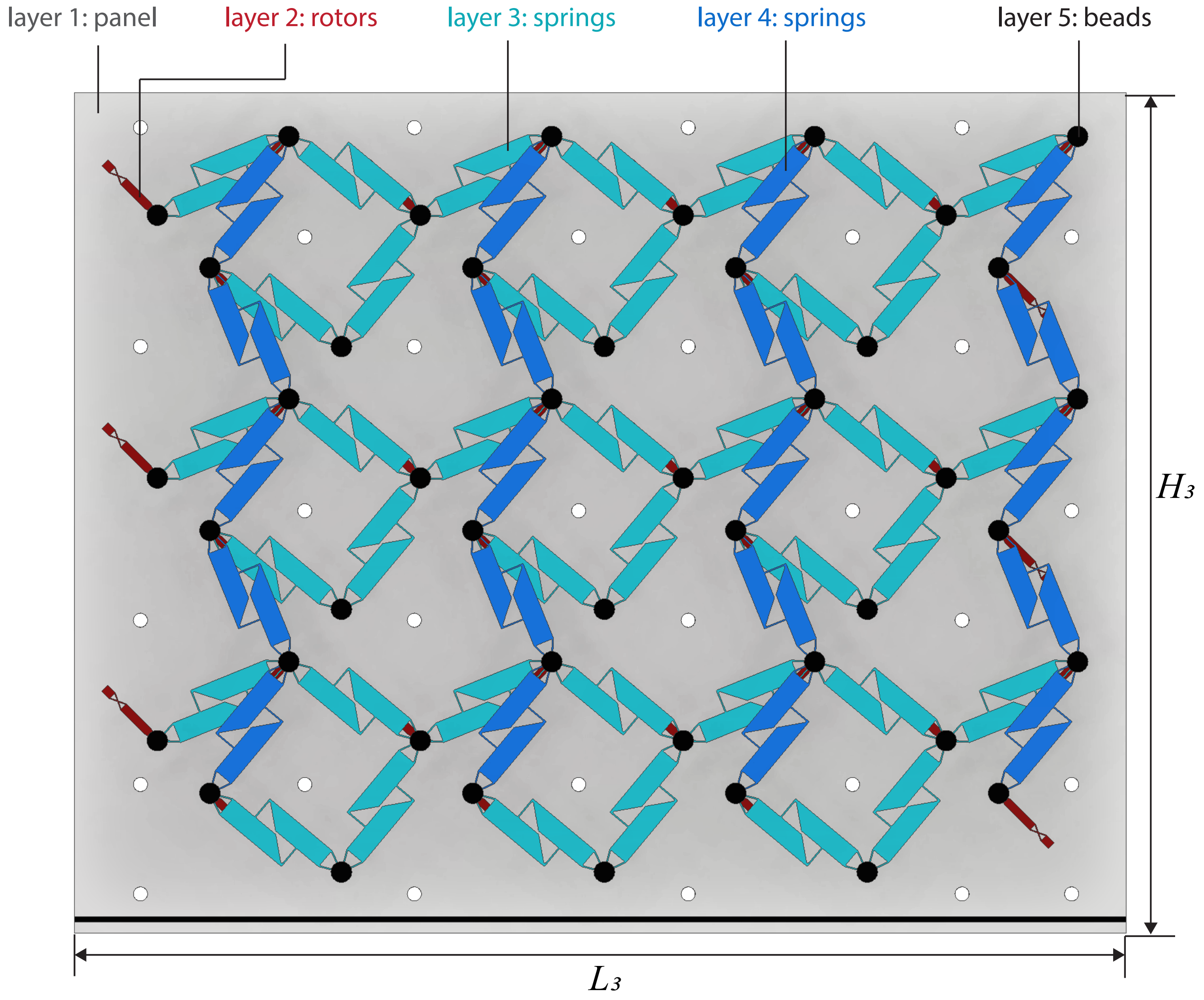

Fig. S22. Design of the two-dimensional topological mechanical metamaterial.

Movie S1. Vertical vibration of the presented 2D metamaterial. a corner floppy mode is observed as the first mode at 88 Hz.

References

1. R De Maesschalck, D Jouan-Rimbaud, DL Massart, The mahalanobis distance. *Chemom. intelligent laboratory systems* **50**, 1–18 (2000).
2. CL Kane, TC Lubensky, Topological boundary modes in isostatic lattices. *Nat. Phys.* **10**, 39–45 (2014).
3. A van Beek, *Advanced engineering design: lifetime performance and reliability.* (2019).
4. M Koster, *Constructieprincipes: voor het nauwkeurig bewegen en positioneren.* (1996).
